# Taxonomy of the ant genus *Proceratium* Roger (Hymenoptera, Formicidae) in the Afrotropical region with a revision of the *P. arnoldi* clade and description of four new species

**DOI:** 10.3897/zookeys.447.7766

**Published:** 2014-10-16

**Authors:** Francisco Hita Garcia, Peter G. Hawkes, Gary D. Alpert

**Affiliations:** 1 Entomology, California Academy of Sciences, 55 Music Concourse Drive, San Francisco, CA 94118, USA; 2 AfriBugs CC, 341 27th Avenue, Villieria, Pretoria, Gauteng Province, 0186, South Africa / Department of Zoology and Entomology, University of Pretoria, Pretoria, Gauteng Province, 0002, South Africa; 3 Entomology Department, Museum of Comparative Zoology, Harvard University, 26 Oxford Street, Cambridge, MA 02138, USA

**Keywords:** Arabuko Sokoke Forest, Gorongosa National Park, Kenya, Mozambique, Nilo Forest, *Proceratium arnoldi* clade, *Proceratium stictum* clade, *Proceratium toschii* clade, Sali Forest, Tanzania, taxonomy

## Abstract

The taxonomy of the genus *Proceratium* Roger is updated for the Afrotropical region. We give an overview of the genus in the region, provide an illustrated identification key to the three clades (*Proceratium arnoldi*, *Proceratium stictum* and *Proceratium toschii* clades) and revise the *Proceratium arnoldi* clade. Four new species from the *Proceratium arnoldi* clade are described as new: *Proceratium sokoke* **sp. n.** from Kenya, *Proceratium carri* **sp. n.** from Mozambique, and *Proceratium nilo* **sp. n.** and *Proceratium sali* **sp. n.** from Tanzania. In order to integrate the new species into the existing taxonomic system we present an illustrated identification key to distinguish the seven Afrotropical species of the *Proceratium arnoldi* clade. In addition, we provide accounts for all members of the *Proceratium arnoldi* clade including detailed descriptions, diagnoses, taxonomic discussions, distribution data and high quality montage images.

## Introduction

The ant genus *Proceratium* Roger, 1863 contains 77 extant and 5 fossil species and is patchily distributed throughout all biogeographical regions (Baroni Urbani and de Andrade 2003; Bolton 2014). Despite this global distribution, these ants are seldom collected, likely due to their cryptobiotic lifestyle (Baroni Urbani and de Andrade 2003). In addition, the natural history of this genus is known from only a few fragmentary reports based on a minority of the known species. At present, it seems that *Proceratium*, like *Discothyrea* Roger, are specialised predators of arthropod eggs. Brown (1958a, 1974, 1980) repeatedly reported several species (*Proceratium avium* Brown, 1974, *Proceratium micrommatum* (Roger, 1863), *Proceratium pergandei* (Emery, 1895) and *Proceratium silaceum* Roger, 1863) carrying, storing and feeding on spider eggs. More recently, Fisher (2005b) also observed the same behaviour and diet in *Proceratium avium* from Mauritius. Most species seem to nest in the soil, below leaf litter, in rotten wood, under deep-set stones, or in tree branches (Brown 1958a, 1974; Baroni Urbani and de Andrade 2003; Fisher 2005b). Colonies of *Proceratium* seem to be relatively small, mostly containing fewer than 100 workers (Brown 1958a, 1958b; Leston 1971), but can have a few hundred workers in some species (Onoyama and Yoshimura 2002; Fisher 2005b). Fisher (2005b) documented the largest colony encountered so far with ca. 350 workers for *Proceratium avium* on Mauritius.

The taxonomy of the genus is in a relatively good condition since Baroni Urbani and de Andrade (2003) revised it on a global scale and provided a morphology-based phylogeny. However, due to the rarity of collections and specimens, there is very little information about intra- and interspecific variation. This becomes apparent from the fact that more than half of the species are known from only one or two type specimens and most of the others have never been collected as a nest series, especially in the tropics. A few species have been discovered and described since 2003 (Fisher 2005b; Xu 2006) and more species can be expected in the future. In their revision, Baroni Urbani and de Andrade (2003) recognised six Afrotropical species belonging to three clades: the *arnoldi* clade with three species (*Proceratium arnoldi* Forel, 1913, *Proceratium burundense* de Andrade, 2003 and *Proceratium lunatum* Terron, 1981), the *Proceratium stictum* clade with one species (*Proceratium boltoni* Leston, 1971) and the *Proceratium toschii* clade with two species (*Proceratium terroni* Bolton, 1995 and *Proceratium toschii* (Consani, 1951)).

In this study we describe four new species: *Proceratium carri* sp. n. from Mozambique, *Proceratium nilo* sp. n. and *Proceratium sali* sp. n. from Tanzania and *Proceratium sokoke* sp. n. from Kenya. We place these four new species in the *Proceratium arnoldi* clade sensu Baroni Urbani and de Andrade (2003), which increases the diversity of the clade in the Afrotropical region to seven species. To separate these from the Afrotropical members of the *Proceratium stictum* and *Proceratium toschii* clades we provide an illustrated identification key to the three clades and we present an illustrated identification key to the seven species of the *Proceratium arnoldi* clade. We also provide species accounts for all members of the clade with detailed descriptions, diagnoses, distribution data, taxonomic discussions and high quality montage images.

## Abbreviations of depositories

The collection abbreviations mostly follow Evenhuis (2014). The material upon which this study is based is located and/or was examined at the following institutions:

AFRC AfriBugs, Pretoria, Gauteng, South Africa

BMNH The Natural History Museum, London, U.K.

CASC California Academy of Sciences, San Francisco, California, U.S.A.

CIRAD Centre de coopération Internationale en recherche agronomique pour le développement, Montpellier, France

MCZ Museum of Comparative Zoology, Cambridge, Massachusetts, U.S.A.

MHNG Muséum d’Histoire Naturelle de la Ville de Genève, Geneva, Switzerland

MNHN Muséum National d’Histoire Naturelle, Paris, France

SAMC Iziko Museums of South Africa, Cape Town, South Africa

## Material and methods

The material for the new species on which this study is based was collected during several recent, still on-going ant diversity inventories in Kenya, Tanzania and Mozambique carried out independently by the three authors. The material from the new species and most of the previously known specimens can be uniquely identified with specimen-level barcodes affixed to each pin (*e.g.* MCZ-ENT00517081 or CASENT0235688). The series of stacked digital colour images were created either by a Canon 7D camera attached to a Leica MZ16 stereomicroscope and source images processed using Helicon Focus 5.3, or with a Leica DFC 425 camera in combination with the Leica Application Suite software. These images are all available online and can be seen on AntWeb (www.antweb.org) and AntWiki (www.antwiki.org).

The measurements were taken with a Leica MZ16 stereomicroscope equipped with an orthogonal pair of micrometers at a magnification of 100×. Measurements and indices are presented as minimum and maximum values and all measurements are expressed in mm to two decimal places. The measurements and indices used in this study are based on Ward (1988), Snelling and Cover (1992) and Baroni Urbani and de Andrade (2003); a few measurements have been redefined following Hita Garcia and Fisher (2011) and we define a few measurements and indices new to *Proceratium* (OI, DPeI and ASI):

EL Eye length – maximum length of eye measured in oblique lateral view

HL Head length – maximum measurable distance from the mid-point of the anterior clypeal margin to the mid-point of the posterior margin of head, measured in full-face view. Impressions on anterior clypeal margin and posterior head margin reduce head length

HLM Head length with mandibles – maximum head length in full-face view including closed mandibles

HW Head width – maximum head width directly behind the eyes, measured in full-face view

HFeL Hind femur length – maximum length of hind femur measured along its external face

HTiL Hind tibia length – maximum length of hind tibia measured on its external face

HBaL Hind basitarsus length – maximum length of hind basitarsus measured along its external face

LT3 Abdominal tergum III length – maximum length of abdominal tergum III (= length of segment III) in lateral view

LS4 Abdominal sternum IV length – maximum length of abdominal sternum IV following Ward (1988)

LT4 Abdominal tergum IV length – maximum length of abdominal tergum IV following Ward (1988)

PeL Petiolar length – maximum length of the petiole in dorsal view including its anterior prolongation

PeW Petiolar width – maximum width of petiole measured in dorsal view

SL Scape length – maximum length of scape shaft excluding basal condyle

TL Total body length – combined length of HLM + WL + PeL + LT3 + LT4

WL Weber’s length – diagonal length of mesosoma in lateral view from the anterior-most point of pronotal slope (excluding neck) to posteroventral margin of propodeal lamella or lobe.

CI Cephalic index – HW / HL × 100

OI Ocular index – EL / HW × 100

SI Scape index – SL / HL × 100

DPeI Dorsal petiole index – PeW / PeL × 100

ASI Abdominal segment index – LT4 /LT3 × 100

IGR Gastral reflexion index – LS4 / LT4

The morphological terminology used in this study follows Snelling and Cover (1992) and Baroni Urbani and de Andrade (2003) with few exceptions. The use of postpetiole, gastral segments and abdominal segments in Baroni Urbani and de Andrade (2003) is confusing at times. To avoid this we do not use the terms postpetiole and gaster and instead use abdominal segment III for the postpetiole following Fisher (2005b) and abdominal segment IV for the gastral segment I. Also, instead of “spur of foretibia”, as in Baroni Urbani and de Andrade (2003), we use the term “calcar of strigil” following Keller (2011). Furthermore, in order to adequately describe pubescence and pilosity we follow Wilson (1955) and use the terms “erect”, “suberect”, “subdecumbent”, “decumbent” and “appressed”. The terminology for the description of surface sculpturing is based on Harris (1979).

We have not been able to examine the palp formula of most species treated in this study. Due to the lack of material we did not want to risk damaging the few specimens (most of them unique types) by moving heads or dissecting mouthparts. Baroni Urbani and de Andrade (2003) provided the palp formula 3.2 for *Proceratium arnoldi*, *Proceratium burundense* and *Proceratium lunatum*, and we were able to confirm this for *Proceratium lunatum*.

### Notes on diagnostic characters

In addition to the Afrotropical material treated here we examined a larger number of species from other regions in order to assess general variation within *Proceratium*. Of special importance was the examination of the interspecific and intraspecific variation in general dimensions, the shape of the petiolar node, especially its ventral process, pilosity/pubescence and surface sculpturing. For this purpose we examined the following species, which include several undescribed species from the Malagasy region (AntWeb): *Proceratium angulinode* de Andrade, 2003 [Malaysia]; *Proceratium austronesicum* de Andrade, 2003 [Papua New Guinea]; *Proceratium avium* Brown, 1974 [Mauritius]; *Proceratium banjaranense* de Andrade, 2003 [Malaysia]; *Proceratium crassicorne* Emery, 1895 [U.S.A.]; *Proceratium croceum* (Roger, 1860) [U.S.A.]; *Proceratium deelemani* Perrault, 1981 [Malaysia, Thailand]; *Proceratium diplopyx* Brown, 1980 [Madagascar]; *Proceratium* fhg-alob [Madagascar]; *Proceratium* fhg-beta [Madagascar]; *Proceratium* fhg-elia [Madagascar]; *Proceratium* fhg-mala [Madagascar]; *Proceratium* fhg-seyc [Seychelles]; *Proceratium google* Fisher, 2005b [Madagascar]; *Proceratium papuanum* Emery, 1897 [Papua New Guinea]; *Proceratium sulawense* de Andrade, 2003 [Indonesia]; *Proceratium terraealtae* de Andrade, 2003 [Malaysia]. On the basis of the examination of the above species and despite the paucity of Afrotropical material, we are very confident in using character sets that appear to be stable within other *Proceratium* species from other regions. Furthermore, the species *Proceratium arnoldi*, *Proceratium carri* and especially *Proceratium lunatum* are known from at least two localities that are often several hundreds or even thousands of km apart. Despite these distances, there is little to no observable intraspecific variation, which is suggestive of a fairly high level of conservation of morphological characteristics over relatively long distances. In contrast, *Proceratium sokoke* and *Proceratium nilo* differ more significantly morphologically, yet their type localities are separated by only 220 km.

Nevertheless, to our surprise, one commonly used character for species level diagnostics turned out to be relatively variable. We observed a lot of intraspecific variation in the surface sculpture of several species throughout most regions. This was already pointed out by Fisher (2005b) who found *Proceratium avium* from Mauritius to display noticeable differences in density and depth of surface sculpture. Our study supports this and extends it to the majority of species examined. The differences are not extreme, however, which means that there are never very different types of sculpture in the same species, but the type encountered can vary from very weakly developed and almost absent to very strongly developed, dense and conspicuous. Consequently, surface sculpture is not recommendable as a primary diagnostic character for the separation of the species treated herein or *Proceratium* in general.

Characters that have proven to be comparatively stable at species level are general dimensions of the head and petiole, the shape of the ventral process of the petiole, the relationship between abdominal segments III and IV, eye size and also pilosity and pubescence. Like in most ants, the shape of the head and petiole turned out to be very stable within species-specific ranges and are useful for diagnostics. The relationship between the lengths of abdominal segments III and IV proved to be valuable, too. The development of the eyes is normally not very important in *Proceratium* since most species have very reduced eyes consisting of one ommatidium, a tiny cluster of indistinct flat ommatidia only distinguishable at high magnifications, or no eyes at all, but a few species, such as *Proceratium burundense* de Andrade, 2003, have slightly larger compound eyes. At first glance the use of pilosity/pubescence might seem challenging since most species of *Proceratium* are very hairy and possess different types of hairs throughout their bodies. However, despite some small variation in density, which can also be attributed to specimen processing, we could not observe any significant variability in pilosity/pubescence within species. Especially the lack or presence of abundant, longer, standing pilosity on top of the very dense mat of subdecumbent to decumbent hairs seems to be species-specific.

## Synopsis of Afrotropical *Proceratium* species

***Proceratium arnoldi* clade**

*Proceratium arnoldi* Forel, 1913 [South Africa, Zimbabwe]

*Proceratium carri* Hita Garcia, Hawkes & Alpert, sp. n. [Mozambique]

*Proceratium burundense* de Andrade, 2003 [Burundi]

*Proceratium lunatum* Terron, 1981 [Cameroon, Gabon, Uganda]

*Proceratium nilo* Hita Garcia, Hawkes & Alpert, sp. n. [Tanzania]

*Proceratium sali* Hita Garcia, Hawkes & Alpert, sp. n. [Tanzania]

*Proceratium sokoke* Hita Garcia, Hawkes & Alpert, sp. n. [Kenya]

***Proceratium stictum* clade**

*Proceratium boltoni* Leston, 1971 [Ghana]

***Proceratium toschii* clade**

*Proceratium terroni* Bolton, 1995 [Cameroon]

*Proceratium toschii* (Consani, 1951) [Kenya]

### Notes on the genus in the Afrotropical region

Considering the biogeography of the genus in sub-Saharan Africa and the rarity of collections, the available data about the distribution patterns of most species is very limited. This is especially true for more than half of the species that are only known from the type locality (*Proceratium burundense*, *Proceratium nilo*, *Proceratium sali*, *Proceratium sokoke*, *Proceratium terroni* and *Proceratium toschii*). The data for the other four species (*Proceratium arnoldi*, *Proceratium boltoni*, *Proceratium carri*, and *Proceratium lunatum*) is a bit better, even though they are also only known from a few localities each. The widest known distribution is seen in *Proceratium lunatum*, which occurs in Cameroon and Gabon, but is also found in Uganda. We expect that this species will also be found in the rainforests of the Democratic Republic of Congo, Congo, and Central African Republic with further sampling in these countries. Leston (1971) noted that for the three then known species (*Proceratium arnoldi*, *Proceratium boltoni* and *Proceratium toschii*) it seemed as if most of the Afrotropical *Proceratium* prefer drier savannah habitats. Nonetheless, *Proceratium lunatum* and *Proceratium terroni* were later described from rainforests in Cameroon (Terron 1981), and the four new species presented in this study inhabit coastal, montane or sandy forest types. So, it seems as if *Proceratium* can be found in most sub-Saharan habitats with the exception of semi-deserts and deserts.

As mentioned above, due to the cryptobiotic lifestyle and small colony size, it is not easy to collect *Proceratium*, but their rarity might also be due to sampling artefacts. For example, intensive sampling in two localities in Kenya (Kakamega Forest and Mpala Research Centre, listed in Hita Garcia et al. 2013) yielded not a single *Proceratium* worker. However, each study found an unidentified male showing clearly that the genus is present in both localities. Consequently, we expect that intensive sampling in the soil stratum in a wide range of habitats throughout the Afrotropical region will very likely yield additional material of the currently known species and from new, yet unknown forms, as can be seen for the Malagasy region, where more than ten undescribed species have been collected within the last decade due to very intensive sampling efforts to collect subterranean ants in general (Fisher 2005a; Yoshimura and Fisher 2014; FHG, unpublished data).

The knowledge on natural history or behaviour for the ten Afrotropical species is extremely limited. Leston (1971) provided the only available data on any of them. He collected a relatively small colony (one queen with 42 workers and a few larvae and pupae) of *Proceratium boltoni* in a piece of rotten wood in the ground and he was also able to collect the same species around 600 m away in the topsoil at the base of a tree. He did not mention more on its natural history except that one greenish dipterous egg and one live nematode were with the colony.

### Identification key to Afrotropical *Proceratium* clades

The following key is derived from Baroni Urbani and de Andrade (2003). It should be noted however, that we exclude the Malagasy species (Madagascar plus the surrounding islands of the Southwest Indian Ocean) from this key. We consider the Malagasy region as a distinct biogeographical region of its own following Bolton (1994).

**Table:** 

1	Clypeus well developed, clearly protruding anteriorly, surrounding the antennal sockets and medially impressed; vertex in full face view weakly concave; calcar of strigil with a basal spine (Fig. 1A, B)	***Proceratium stictum* clade**
–	Clypeus reduced, only slightly protruding anteriorly, not surrounding the antennal sockets and not medially impressed; vertex in full face view not concave, usually weakly to moderately convex; calcar of strigil without basal spine (Fig. 1C, D)	**2**
2	Frontal carinae relatively close to each other, either converging and posteriorly fused (Fig. 2A) or approaching each other medially, almost fusing, but narrowly diverging posteriorly; lower mesopleura flat	***Proceratium toschii* clade**
–	Frontal carinae relatively far from each other, never approaching medially and widely diverging posteriorly; lower mesopleura posteriorly inflated (Fig. 2B)	***Proceratium arnoldi* clade**

**Figure 1.**
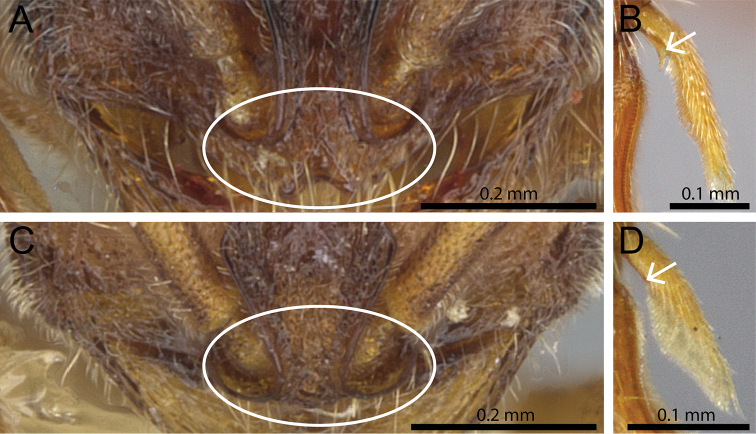
Anterior part of cephalic dorsum in full-face view showing clypeus (within white ellipse) and calcar of strigil (white arrows indicate position of calcar). **A** *Proceratium boltoni* (CASENT0902424) (Will Ericson 2013) **B** *Proceratium diplopyx* (Malagasy member of *Proceratium stictum* clade) **C** *Proceratium burundense* (CASENT0902427) (Will Ericson 2013) **D** *Proceratium sokoke* (MCZ-ENT00520482).

**Figure 2.**
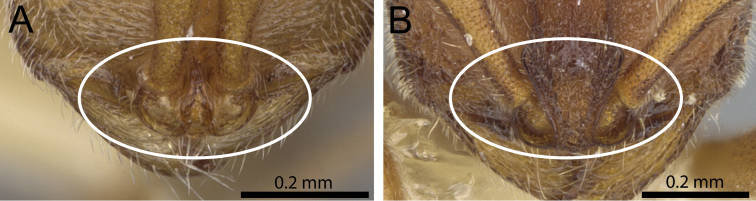
Anterior part of cephalic dorsum in full-face view showing frontal carinae (within white ellipse). **A** *Proceratium terroni* (CASENT0914223) (Michele Esposito 2014) **B** *Proceratium burundense* (CASENT0902427) (Will Ericson 2013).

### The *Proceratium arnoldi* clade

**Diagnosis.** The following diagnosis is based on Baroni Urbani and de Andrade (2003): clypeus reduced, only slightly protruding anteriorly, not surrounding the antennal sockets and not medially impressed; frontal carinae widely separated, not approaching each other closely and strongly diverging posteriorly; pair of transparent maculae on vertexal angles present in all but one species; calcar of strigil without basal spine; bulla usually located medially at the posterior end of the third abdominal segment; lower mesopleura posteriorly inflated.

**Notes.** Baroni Urbani and de Andrade (2003) gave the presence of transparent maculae on the vertexal angles and a bulla located medially at the posterior end of the third abdominal segment as characteristic of the clade. Nevertheless, the maculae are not present in all five species. Actually, in the new species *Proceratium carri* there is not a trace of maculae on the vertexal angles, whereas all other clade members (including the eighth species of the clade *Proceratium galilaeum* de Andrade from Israel) possess very conspicuous maculae. The holotype of *Proceratium arnoldi* seemed to lack the maculae at first sight, but closer examination under higher magnifications and different light settings revealed them later. The other clade-specific character, the bulla on the third abdominal segment, is indeed present in all species of the *Proceratium arnoldi* clade, even though it is much less developed in *Proceratium arnoldi* and *Proceratium carri* than in the remainder of the clade. Nevertheless, even though these characters are not always fully developed, the seven Afrotropical species of the *Proceratium arnoldi* clade can be easily distinguished from the single Afrotropical species of the *Proceratium stictum* clade and the two species of the *Proceratium toschii* clade with the diagnosis given above.

### Identification key to Afrotropical species of *Proceratium arnoldi* clade

**Table:** 

1	No maculae present on vertexal angles of head (Fig. 3A); abdominal segment IV relatively long, around 1.6 times longer than abdominal segment III (ASI 156–159) (Fig. 3D) [Mozambique]	***Proceratium carri***
–	Maculae on vertexal angles of head present and usually very conspicuous, but sometimes difficult to see (Fig. 3B, 3C); abdominal segment IV always conspicuously shorter than above, between 1.0 to 1.3 times longer than abdominal segment III (ASI 102–132) (Fig. 3E, F)	**2**
2	Eyes absent (OI 0) (Fig. 4A) [Tanzania]	***Proceratium nilo***
–	Eyes variable in size, but always present (OI 3–8) (Fig. 4B, C)	**3**
3	Eyes larger (OI 8) (Fig. 5A); ventral process of petiole with posteroventral corner conspicuously projecting ventrally, almost spiniform (Fig. 5D) [Burundi]	***Proceratium burundense***
–	Eyes always smaller (OI 3–5) (Fig. 5B, C); ventral process of petiole usually more or less rectangular without posteroventral corner conspicuously projecting ventrally, rarely posteroventral corner weakly pointing posteroventrally, but distinctly less conspicuously than above (Fig. 5E, F)	**4**
4	Head, mesosoma and petiole with numerous long, fine, suberect to erect hairs on top of dense mat of much shorter decumbent to subdecumbent pubescence (Fig. 6A, B)	**5**
–	Head, mesosoma and petiole with mat of short decumbent to subdecumbent pubescence only, without numerous longer, fine suberect to erect hairs (Fig. 6C), sometimes parts of pubescence on mesosoma suberect to erect (Fig. 6D)	**6**
5	Petiolar node in profile appearing thicker and stouter, with lower third of anterior face strongly produced anteriorly (Fig. 7A) [Kenya]	***Proceratium sokoke***
–	Petiolar node higher and thinner than above, more strongly anteroposteriorly compressed, with anterior face relatively straight (Fig. 7B) [Tanzania]	***Proceratium sali***
6	Head relatively longer (CI 85–87) (Fig. 8A); surface sculpture weak and superficial; body colouration yellow to very light brown (Fig. 8B) [South Africa, Zimbabwe]	***Proceratium arnoldi***
–	Head relatively shorter (CI 92–95) (Fig. 8C); surface sculpture better developed, usually deeply foveolate; body colouration of darker brown than above (Fig. 8D) [Cameroon, Gabon and Uganda]	***Proceratium lunatum***

**Figure 3.**
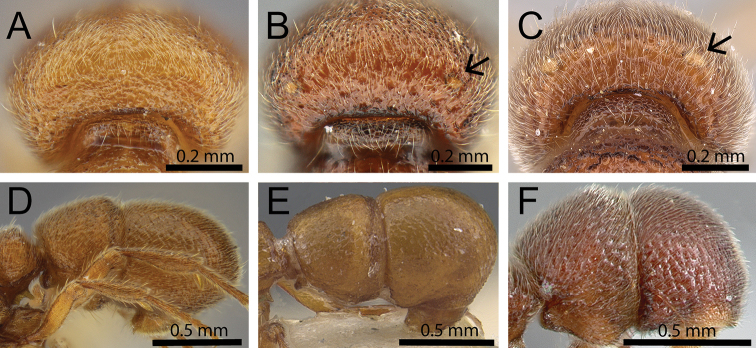
Vertex in posterodorsal view (black arrows indicate maculae) and abdominal segments III and IV in profile. **A, D** *Proceratium carri* (MCZ-ENT00517081) **B** *Proceratium sokoke* (MCZ-ENT00520482) **C** *Proceratium sali* (CASENT0235689) (Will Ericson 2011) **E** *Proceratium arnoldi* (CASENT0907203) (Will Ericson 2013) **F** *Proceratium lunatum* (CASENT0005926).

**Figure 4.**
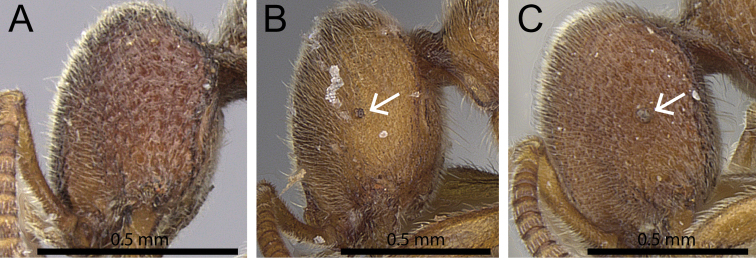
Head in profile showing eye (white arrows indicate location of eye, if present). **A** *Proceratium nilo* (CASENT0235688) (Will Ericson 2011) **B** *Proceratium arnoldi* (CASENT0914281) (Michele Esposito 2014) **C** *Proceratium burundense* (CASENT0902427) (Will Ericson 2013).

**Figure 5.**
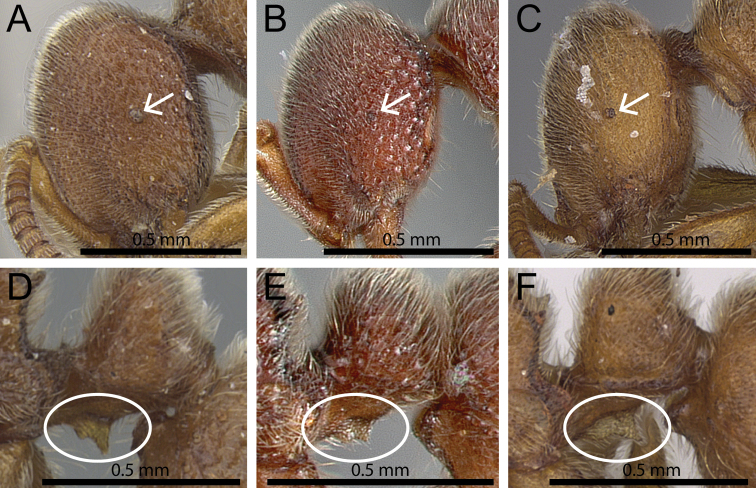
Head in profile showing eye (white arrows indicate location of eye) and petiole with ventral process in profile (within white ellipse). **A, D** *Proceratium burundense* (CASENT0902427) (Will Ericson 2013) **B, E** *Proceratium lunatum* (CASENT0005926) **C, F** *Proceratium arnoldi* (CASENT0914281) (Michele Esposito 2014).

**Figure 6.**
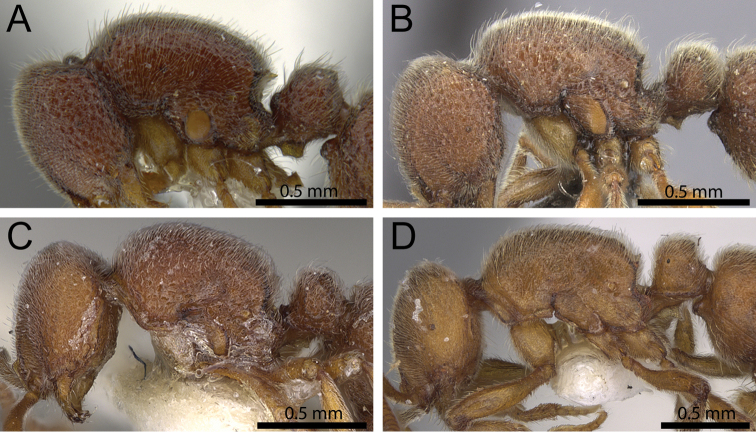
Head, mesosoma and petiole in profile. **A** *Proceratium sokoke* (MCZ-ENT00520482) **B** *Proceratium sali* (CASENT0235689) (Will Ericson 2011) **C** *Proceratium lunatum* (CASENT0914221) (Michele Esposito 2014) **D** *Proceratium arnoldi* (CASENT0914281).

**Figure 7.**
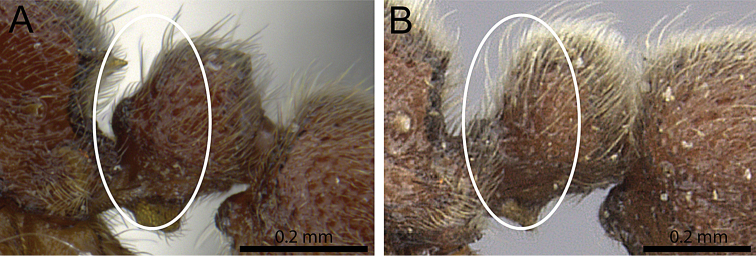
Petiole in profile (anterior face within white ellipse). **A** *Proceratium sokoke* (MCZ-ENT00520482) **B** *Proceratium sali* (CASENT0235689) (Will Ericson 2011).

**Figure 8.**
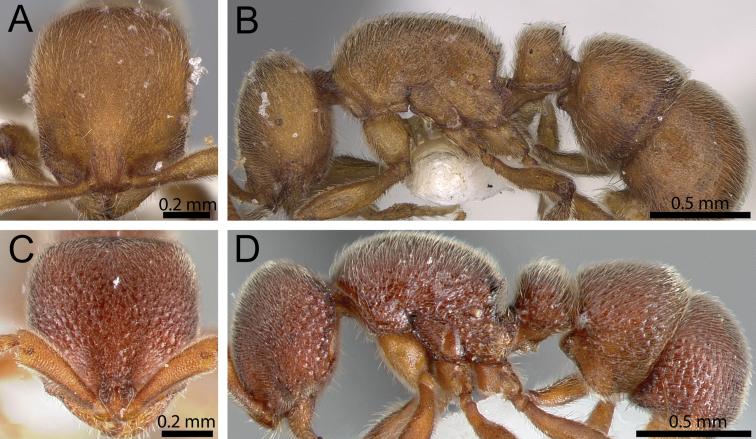
Head in full-face view and body in profile. **A, B** *Proceratium arnoldi* (CASENT0914281) (Michele Esposito 2014) **C, D** *Proceratium lunatum* (CASENT0005926).

## Review of species

### Proceratium arnoldi

Taxon classificationAnimaliaHymenopteraFormicidae

Forel, 1913

**Figure 9.**
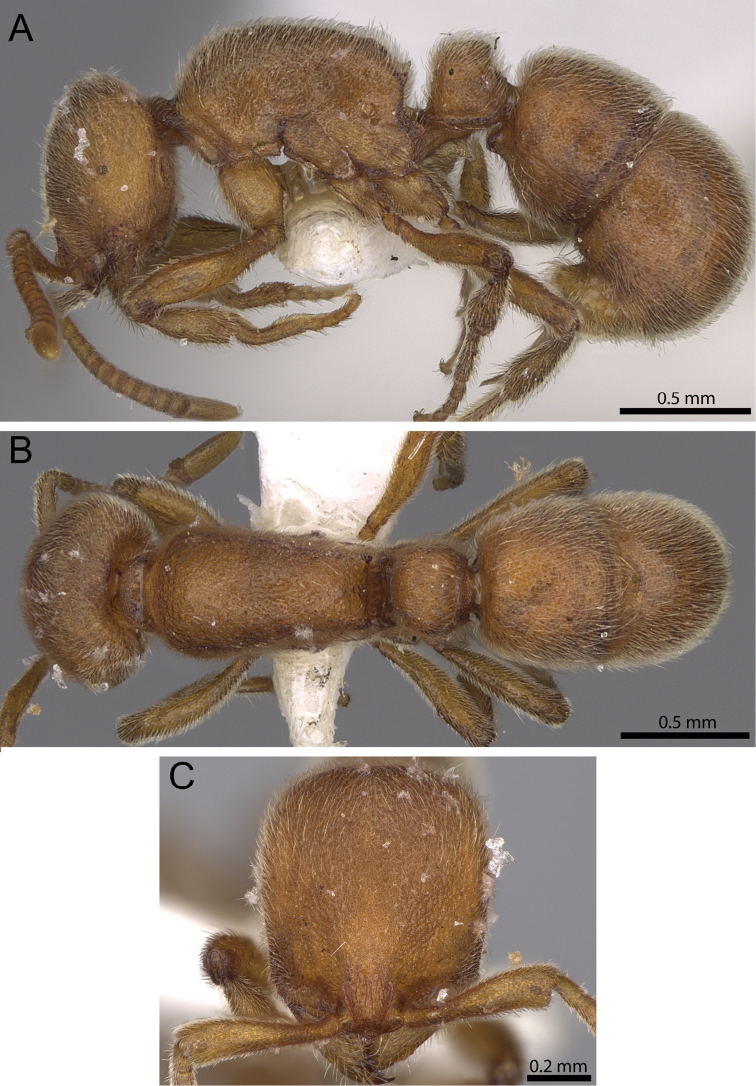
*Proceratium arnoldi* worker (CASENT0914281) (Michele Esposito 2014). **A** Body in profile **B** Body in dorsal view **C** head in full-face view.

Figs 3E , 4B , 5C , 5F , 6D , 8A , 8B , 9A , 9B , 9C , 18

Proceratium (Sysphincta) arnoldi Forel, 1913: 210. [Combination in *Sysphincta*: Arnold 1915: 35; in *Proceratium*: Brown 1958b: 247; see also: Baroni Urbani and de Andrade 2003: 297]

#### Type material.

**Holotype**, pinned worker, ZIMBABWE, Bulawayo, (MHNG: CASENT0907203).

[Note: There are two known “type” specimens of *Proceratium arnoldi*, and there is some confusion about their labels and type status. De Andrade (in Baroni Urbani and de Andrade 2003) stated that the specimen from BMNH (CASENT0902425) labelled as syntype with the collection data “Bulawayo, S. Rhodesia, 29.III.1913, (*G. Arnold*)” is likely the specimen on which Arnold (1915) based his re-description and was probably never examined by Forel, even though it seems that both specimens belong to the same collection. We agree with de Andrade that this is not a type specimen].

#### Non-type material.

SOUTH AFRICA: Natal, N of Richard’s Bay, 28.40S, 32.14E, 26.I.–11.II.1991 (*A. de Kock & J.D. Majer*) (BMNH: CASENT0914281); ZIMBABWE: Bulawayo, S. Rhodesia, 29.III.1913, (*G. Arnold*) (BMNH: CASENT0902425).

#### Diagnosis.

The following character combination distinguishes *Proceratium arnoldi* from the other Afrotropical members of the *Proceratium arnoldi* clade: eyes very small, consisting of a single dark ommatidium (OI 3–5); head clearly longer than broad (CI 85–87); maculae on vertexal angles of head well developed and conspicuous; mesopleurae weakly to moderately inflated posteriorly; petiolar node high nodiform, anteroposteriorly compressed, with anterior face relatively straight; petiole in dorsal view between 1.0 and 1.2 times wider than long (DPeI 106–114); ventral process of petiole lamelliform, subrectangular, anteroventral corner blunt and posteroventral corner conspicuously projecting posteroventrally; abdominal segment IV around 1.2 to 1.3 times longer than abdominal segment III (ASI 116–132); head, mesosoma and petiole with mat of short decumbent to subdecumbent pubescence only, without any longer, fine suberect to erect hairs.

#### Worker measurements

**(N=5).** TL 3.27–3.56; EL 0.02–0.03; SL 0.49–0.52; HL 0.79–0.84; HLM 0.94–1.02; HW 0.69–0.71; WL 0.91–1.03; HFeL 0.59–0.67; HTiL 0.48–0.51; HBaL 0.38–0.46; PeL 0.33–0.34; PeW 0.35–0.38; DPeI 106–114; LT3 0.47–0.54; LS4 0.24–0.25; LT4 0.62–0.64; OI 3–5; CI 85–87; SI 60–63; IGR 0.38–0.41; ASI 116–132.

#### Worker description.

In full-face view head clearly longer than broad (CI 85–87), sides weakly convex, gently broadening posteriorly, vertex flat to weakly convex. Clypeus medially reduced, its anterior margin subconvex to slightly triangular, only slightly protruding anteriorly, not surrounding the antennal sockets and not medially impressed, antennal socket with broad torulus. Frontal carinae relatively very short and widely separated, not converging medially and strongly diverging posteriorly, partially covering antennal insertions; frontal carinae conspicuously raised anteriorly, much less so posteriorly. Eyes very small, consisting of one to four weak ommatidia (OI 5) and located on mid line of head. Mandibles elongate-triangular; masticatory margin of mandibles with one well developed apical tooth and a series of four denticles decreasing in size towards basal-most denticle. Mesosoma weakly to moderately convex in profile and approximately as long as the maximum head length including mandibles. Lower mesopleurae with well impressed sutures, no other sutures developed on lateral or dorsal mesosoma, mesopleurae weakly to moderately inflated posteriorly; propodeum in profile armed with very small, pointed or blunt teeth, propodeal lobes weakly to moderately developed, lamellate, subtriangular and blunt; declivitous face of propodeum between teeth and lobes concave; in posterodorsal view sides of propodeum separated from declivitous face by margin connecting propodeal lobes and propodeal teeth. Legs slender and elongate; pro- and mesotibiae with pectinate spurs; calcar of strigil without basal spine. Petiolar node in profile high, blocky nodiform, anterior face of petiole relatively straight, anterior and posterior faces approximately parallel, dorsum of node flat to very weakly convex; petiole in dorsal view between 1.0 and 1.2 times wider than long (DPeI 106–114), petiolar node in dorsal view clearly much broader than long; ventral process of petiole lamelliform, subrectangular, anteroventral corner blunt and posteroventral corner conspicuously projecting posteroventrally. In dorsal view abdominal segment III anteriorly broader than petiole; its sides diverging posteriorly; dorsum of abdominal tergum III with posteromedial, weakly developed, semitransparent, flat bulla below the integument; abdominal sternite III anteromedially with a marked subtriangular projection. Constriction between abdominal segment III and IV conspicuously impressed. Abdominal segment IV strongly recurved (IGR 0.38–0.41), conspicuously rounded on its curvature, especially posteriorly, abdominal tergum IV around 1.2 to 1.3 times longer than abdominal segment III (ASI 116–132); small, faint and semitransparent bulla situated posteromedially on abdominal tergum IV; remaining abdominal tergites and sternites relatively inconspicuous and curved ventrally. Whole body covered with dense mat of relatively short, decumbent to suberect pubescence without any abundant, much longer, suberect to erect, long, fine, standing hairs. Mandibles longitudinally rugose; most of body irregularly foveolate and/or punctate, sculpture best developed on cephalic dorsum, much weaker on remainder of body, especially weak, almost smooth on abdominal segments III and IV. Body colour uniformly yellowish to light brown.

#### Distribution and ecology.

At present, *Proceratium arnoldi* is only known from two localities in Zimbabwe and South Africa (Fig. 18). These localities are relatively far apart, but we expect that the species is more widespread and will be collected in the area between. Despite that it was described more than 100 years ago, there is no information available about its natural history.

#### Taxonomic notes.

*Proceratium arnoldi* is well recognisable within the clade. Its relatively thin head in full-face view (CI 85–87) groups it close to *Proceratium carri* (CI 85–86) and separates both from the other five species that have thicker heads (CI 91–95). However, *Proceratium carri* is not likely to be confused with *Proceratium arnoldi*. The latter possesses a mat of short decumbent to subdecumbent pubescence but without numerous much longer, fine standing hairs. These hairs are present in *Proceratium carri*, which also has a much longer abdominal segment IV in relation to abdominal segment III (ASI 156–159) than *Proceratium arnoldi* (ASI 116–132).

#### Variation.

We only observed some minor, very normal variation in body size in the known material of *Proceratium arnoldi* with the specimens from South Africa being somewhat larger (WL 0.98–1.03) than the ones from Zimbabwe (WL 0.91–0.92). Otherwise, there is no observable intraspecific variation.

### Proceratium burundense

Taxon classificationAnimaliaHymenopteraFormicidae

de Andrade, 2003

**Figure 10.**
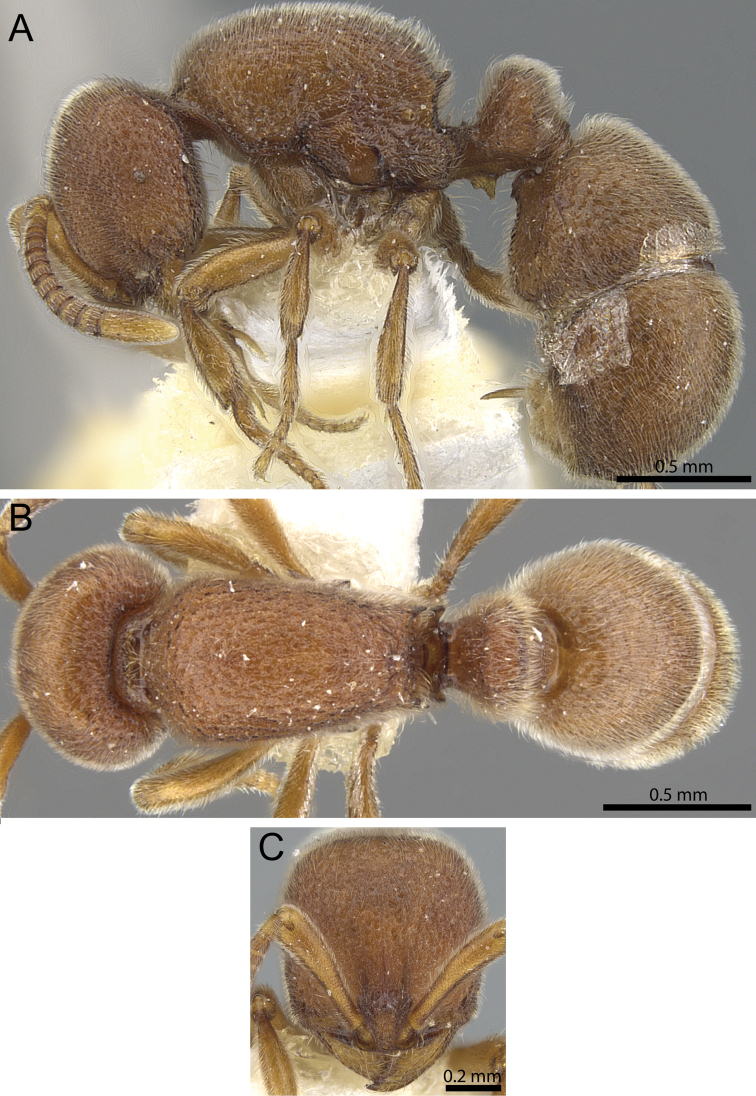
*Proceratium burundense* holotype worker (CASENT0902427) (Will Ericson 2013). **A** Body in profile **B** Body in dorsal view **C** head in full-face view.

Figs 1C , 2B , 4C , 5A , 5D , 10A , 10B , 10C , 18

Proceratium burundense de Andrade, in Baroni Urbani and de Andrade 2003: 294.

#### Type material.

**Holotype**, pinned worker, BURUNDI, Bujumbura, 4.III.77, (*A. Dejean*) (BMNH: CASENT0902427).

#### Diagnosis.

*Proceratium burundense* is easily distinguishable from the other Afrotropical species of the *Proceratium arnoldi* clade by the following character combination: eyes larger, consisting of nine well developed ommatidia (OI 8); head slightly longer than broad (CI 91); maculae on vertexal angles of head well developed and conspicuous; mesopleurae moderately inflated posteriorly; petiolar node high nodiform, anteroposteriorly compressed, with anterior face relatively straight; petiole around 1.2 times wider than long (DPeI 121); ventral process of petiole lamelliform and subrectangular with posteroventral corner strongly pointing ventrally, almost spiniform; abdominal segment IV less than 1.1 times longer than abdominal segment III (ASI 106); head, mesosoma and petiole with mat of short decumbent to subdecumbent pubescence only, without any longer, fine suberect to erect hairs.

#### Worker measurements

**(N=1).** TL 3.44; EL 0.06; SL 0.54; HL 0.79; HLM 0.90; HW 0.72; WL 1.02; HFeL 0.59; HTiL 0.51; HBaL 0.39; PeL 0.32; PeW 0.39; DPeI 121; LT3 0.58; LS4 0.24; LT4 0.61; OI 8; CI 91; SI 0.68; IGR 0.39; ASI 106.

[Note: the singleton holotype was examined in BMNH, but not measured. The measurements presented above are the ones given by Baroni Urbani and de Andrade (2003) except for HLM, PeL, PeW and LT3, which were measured from the montage images of the specimen]

#### Worker description.

In full-face view head slightly longer than broad (CI 91), sides weakly convex, not broadening posteriorly, vertex flat to weakly convex. Clypeus medially reduced, its anterior margin subconvex to slightly triangular, only slightly protruding anteriorly, not surrounding the antennal sockets and not medially impressed, antennal socket with broad torulus. Frontal carinae relatively short and widely separated, not converging medially and strongly diverging posteriorly, partially covering antennal insertions; frontal carinae conspicuously raised on their anterior half, much less posteriorly. Eyes small (but larger than in remainder of group), consisting of nine well developed ommatidia (OI 8) and located on mid line of head. Mandibles elongate-triangular; masticatory margin of mandibles with four to five relatively small teeth/denticles, decreasing in size from larger apical tooth to very small basal denticle. Mesosoma clearly convex in profile and slightly longer than maximum head length including mandibles. Lower mesopleurae with well impressed sutures, no other sutures developed on lateral or dorsal mesosoma; mesopleurae moderately inflated posteriorly; propodeum in profile armed with small, pointed teeth, propodeal lobes well developed, lamellate, subtriangular and blunt; declivitous face of propodeum between teeth and lobes noticeably concave; in posterodorsal view sides of propodeum separated from declivitous face by margin connecting propodeal lobes and propodeal teeth. Legs slender and elongate; pro- and mesotibiae with pectinate spurs; calcar of strigil without basal spine. Petiolar node in profile high, blocky nodiform, anterior face of petiole relatively straight, anterior and posterior faces approximately parallel, dorsum of node flat to weakly convex; petiole in dorsal view around 1.2 times wider than long (DPeI 121), petiolar node in dorsal view clearly much broader than long; ventral process of petiole lamelliform and subrectangular with posteroventral corner strongly pointing ventrally, almost spiniform. In dorsal view abdominal segment III anteriorly broader than petiole; its sides diverging posteriorly; dorsum of abdominal tergum III with posteromedial, very conspicuous, semitransparent, flat bulla below the integument; abdominal sternite III anteromedially with a marked subtriangular projection. Constriction between abdominal segment III and IV conspicuously impressed. Abdominal segment IV strongly recurved (IGR 0.39), conspicuously rounded on its curvature, especially posteriorly, abdominal tergum IV only slightly longer than abdominal segment III (ASI 106); semitransparent bulla situated posteromedially on abdominal tergum IV; remaining abdominal tergites and sternites relatively inconspicuous and curved ventrally. Whole body covered with dense mat of relatively short, decumbent to suberect pubescence without any abundant, much longer, suberect to erect, long, fine, standing hairs. Mandibles longitudinally rugose; most of body irregularly foveolate and/or granulate, sculpture best developed on cephalic and mesosomal dorsum, less so remainder of body and especially weak on most of relatively shining abdominal tergum IV, abdominal tergum IV posteroventrally (shortly before abdominal tergum V) with irregularly rugose area; inflated, posterior part of mesopleura and declivitous face of propodeum also mostly unsculptured and relatively smooth and shining. Head, mesosoma, petiole and remaining abdominal segments brown; mandibles, antennae, and legs of lighter brown.

#### Distribution and ecology.

The species is only known from the type locality in Burundi (Fig. 18). Unfortunately, the label provides very little locality data. Bujumbura is the capital of Burundi, but it is unclear if *Proceratium burundense* was collected in an urban habitat or in the area surrounding of the city. Also, there is no natural history data available.

#### Taxonomic notes.

As noted above, the presence of a larger compound eye that consists of nine well developed ommatidia in the worker caste distinguishes *Proceratium burundense* (OI 8) from the other six species of the clade (OI 0–5), but also from most other known *Proceratium* species that have either no eyes, just one ommatidium or a few very weak, almost indistinguishable ommatidia only visible under higher magnifications (Baroni Urbani and de Andrade 2003). Baroni Urbani and de Andrade (2003) pointed out that they consider the eye of *Proceratium burundense* as the only real compound eye found in workers. It should be mentioned that the known subergatoid intercastes have much larger compound eyes, as is the case in *Proceratium toschii*, but the presence of ocelli separates these immediately from normal workers, which lack ocelli. Not considering eye size, *Proceratium burundense* shares a thicker head (CI 91) in full-face view with *Proceratium nilo*, *Proceratium sali*, *Proceratium lunatum* and *Proceratium sokoke* (CI 91–95), which contrasts with the thinner head seen in *Proceratium arnoldi* and *Proceratium carri* (CI 85–87). In addition, *Proceratium burundense*, as well as *Proceratium arnoldi* and *Proceratium lunatum*, lack numerous long, fine standing hairs on top of a mat of short decumbent to subdecumbent pubescence while these hairs are present in *Proceratium nilo*, *Proceratium sali*, *Proceratium carri* and *Proceratium sokoke*. Furthermore, the ventral process of the petiole, which is subrectangular with the posteroventral corner strongly pointing ventrally, almost spiniform, in *Proceratium burundense* separates it clearly from *Proceratium nilo*, *Proceratium sali*, *Proceratium lunatum* and *Proceratium sokoke* that have a process without a posteroventral corner that is strongly projected ventrally. The shape of the ventral process in *Proceratium arnoldi* and *Proceratium carri* is closest to the one seen in *Proceratium burundense* but the latter species cannot be misidentified with *Proceratium arnoldi* and *Proceratium carri* based on the characters presented above (e.g. head shape, eye size, pilosity). *Proceratium lunatum* is likely the species morphologically closest to *Proceratium burundense* since they share most characters except for eye size, the shape of the ventral process of the petiole, and the propodeal of the propodeal teeth (very small and blunt in *Proceratium lunatum* vs. small but longer and clearly pointed in *Proceratium burundense*.

#### Variation.

Since *Proceratium burundense* is only known from the holotype, there is no information about intraspecific variation.

### Proceratium carri sp. n.

Taxon classificationAnimaliaHymenopteraFormicidae

http://zoobank.org/8DF4FD29-A215-4FF7-AA15-F8C11376A7FD

**Figure 11.**
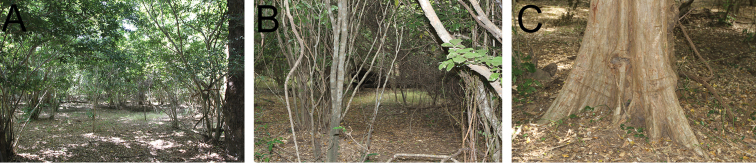
Type locality of *Proceratium carri*. **A** Sandy Forest habitat with moderate canopy cover, little understory and thin leaf litter layer **B** Shady forest understory **C** Tree under which the holotype was collected from soil.

**Figure 12.**
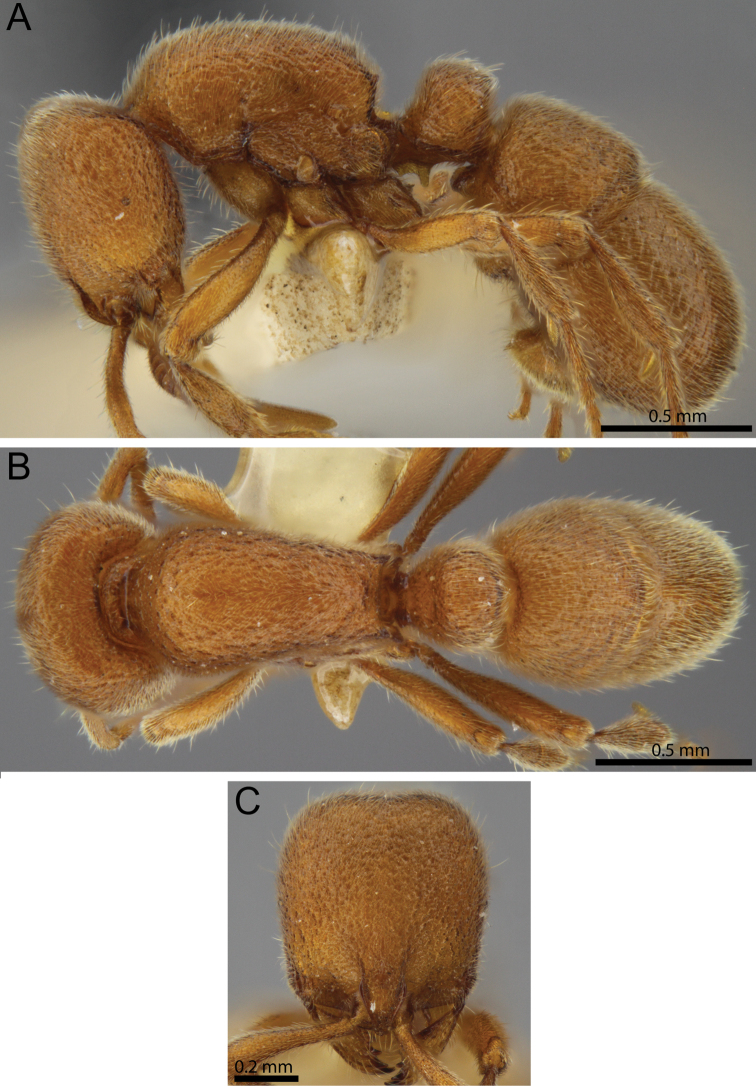
*Proceratium carri* sp. n. holotype worker (MCZ-ENT00517081). **A** Body in profile **B** Body in dorsal view **C** head in full-face view.

**Figure 13.**
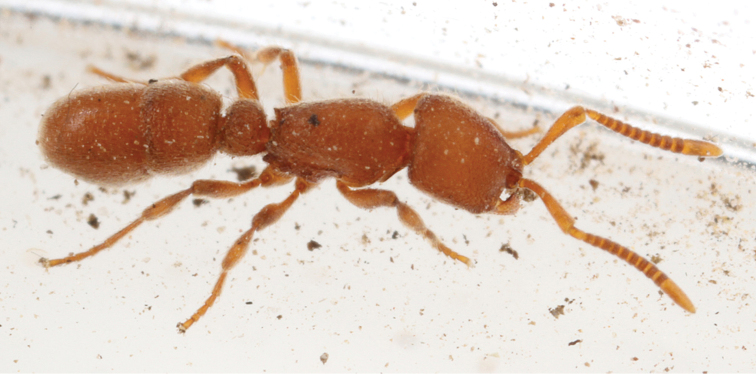
Photo of the living holotype of *Proceratium carri* (MCZ-ENT00517081) taken after discovery in soil sample prior to preservation of the specimen in ethanol.

Figs 3A , 3D , 11A , 11B , 11C , 12A , 12B , 12C , 13 , 18

#### Type material.

**Holotype**, pinned worker, MOZAMBIQUE, Sofala, Gorongosa National Park, 4 km NW of Chitengo, 18°57'34.1"S, 34°20'30.7"E, 41 m, sandy forest on road #2, dry soil-leaf litter, collected three bags of dry soil, misc. ants, WP113, 28.IV.2013 (*G.D. Alpert*) (MCZ: MCZ-ENT00517081).

#### Non-type material.

MOZAMBIQUE: Tete, Moatize, Haul Road 6, 30 km, 15.97644S, 33.8557E, 336 m, closed undifferentiated woodland 13.IV.2014 (*P. Hawkes & R. Mulaudzi*) (AFRC: CASENT0250381), Tete, Moatize, Haul Road 6, 6 km, 15.78187 S, 33.81614 E, 303 m, closed undifferentiated woodland 14.IV.2014 (*P. Hawkes & R. Mulaudzi*) (AFRC: CASENT0250382).

#### Diagnosis.

*Proceratium carri* differs from the other Afrotropical members of the *Proceratium arnoldi* clade by the following character combination: eyes strongly reduced, consisting of a single ommatidium (OI 5); head clearly longer than broad (CI 85–86); maculae on vertexal angles of head absent; mesopleurae weakly to moderately inflated posteriorly; petiolar node high nodiform, anteroposteriorly compressed, with anterior face relatively straight; petiole in dorsal view between 1.1 to 1.2 times wider than long DPeI 111–119; ventral process of petiole lamelliform, subrectangular, lamella weakly pointed anteriorly and strongly pointed posteriorly; abdominal segment IV around 1.6 times longer than abdominal segment III (ASI 156–159); head, mesosoma and petiole with numerous long, fine, suberect to erect hairs on top of dense mat of much shorter decumbent to subdecumbent pubescence.

#### Worker measurements

**(N=2).** TL 2.96–3.07; EL 0.03; SL 0.48–0.51; HL 0.75–0.77; HLM 0.88–0.92; HW 0.63–0.66; WL 0.81–0.82; HFeL 0.55–0.59; HTiL 0.46–0.50; HBaL 0.34–0.35; PeL 0.26–0.29; PeW 0.31–0.32; DPeI 111–119; LT3 0.39–0.41; LS4 0.16–0.18; LT4 0.62–0.64; OI 5; CI 85–86; SI 64–66; IGR 0.25–0.29; ASI 156–159.

#### Worker description.

In full-face view head clearly longer than broad (CI 85–86), sides weakly convex, gently broadening posteriorly, vertex shallowly concave. Clypeus medially reduced, its anterior margin convex to slightly triangular, only slightly protruding anteriorly, not surrounding antennal sockets and not medially impressed, antennal socket with broad torulus. Frontal carinae relatively short and widely separated, not converging medially and strongly diverging posteriorly, partially covering antennal insertions; frontal carinae conspicuously raised on their anterior two thirds, much less posteriorly. Eyes small, consisting of a single ommatidium and located on mid line of head. Antennae 12-segmented, scapes short (SI 64–66), not reaching vertexal margin and noticeably thickening apically, first and last funicular segments longer than broad, remaining funicular segments noticeably broader than long. Mandibles elongate-triangular; masticatory margin of mandibles with five teeth/denticles, decreasing in size from larger apical tooth to basal denticles. Mesosoma weakly to moderately convex in profile and clearly shorter than maximum head length including mandibles. Lower mesopleurae with well impressed sutures, no other sutures developed on lateral or dorsal mesosoma; mesopleurae weakly to moderately inflated posteriorly; propodeum in profile armed with small, pointed teeth, propodeal lobes moderately developed, lamellate and blunt; declivitous face of propodeum between teeth and lobes noticeably concave; in posterodorsal view sides of propodeum separated from declivitous face by margin connecting propodeal lobes and propodeal teeth. Legs slender and elongate; all tibiae with pectinate spur; calcar of strigil without basal spine; pretarsal claws simple; arolia well developed. Petiolar node in profile high, blocky nodiform, anterior face of petiole relatively straight, anterior and posterior faces approximately parallel, dorsum of node flat to weakly convex; petiole in dorsal view between 1.1 to 1.2 times wider than long (DPeI 111–119), petiolar node in dorsal view clearly much broader than long and transverse; ventral process of petiole lamelliform, subrectangular, lamella weakly pointed anteriorly and strongly pointed posteriorly. In dorsal view abdominal segment III anteriorly broader than petiole; its sides diverging posteriorly; dorsum of abdominal tergum III with posteromedial, very faint, semitransparent, flat bulla below the integument; abdominal sternite III anteromedially with a marked subtriangular projection. Constriction between abdominal segment III and IV conspicuously impressed. Abdominal segment IV strongly recurved (IGR 0.25–0.29), conspicuously rounded on its curvature, especially posteriorly, abdominal tergum IV relatively long, around 1.6 times longer than abdominal segment III (ASI 156–159); moderately large, semitransparent and elongate bulla situated posteromedially on abdominal tergum IV; remaining abdominal tergites and sternites relatively inconspicuous and curved ventrally. Whole body covered with dense mat of relatively short, appressed to subdecumbent pubescence; antennal scapes and legs also with moderately abundant, much longer (several times longer than pubescence), subdecumbent to erect, long, fine, standing hairs; head, mesosoma, petiole and abdominal segments with same type of long, standing pilosity, but usually more scattered than on appendages. Mandibles longitudinally rugose; most of body irregularly foveolate and/or punctate, sculpture best developed on cephalic dorsum and abdominal tergum III, less so on sides of mesosoma and especially weak on most of relatively smooth and shining abdominal tergum IV, abdominal tergum IV posteroventrally (shortly before abdominal tergum V) with small irregularly rugose area; inflated, posterior part of mesopleura and declivitous face of propodeum also only very weakly sculptured and relatively smooth and shining. Body of uniformly yellow to light orange brown colour.

#### Etymology.

The name of the new species is a patronym dedicated to the American entrepreneur and philanthropist Gregg C. Carr. We want to honour his great accomplishments for the restoration of the Gorongosa National Park in Mozambique and his efforts in the conservation of African biodiversity.

#### Distribution and ecology.

Gorongosa National Park is geographically divided into two sections, a higher elevation section on Gorongosa Mountain (1863 m summit) with a montane rainforest and a separate lowland elevation (39 m) section within the southern end of the Great African Rift Valley of east Africa (Fig. 18). The holotype of *Proceratium carri* was collected at the lower elevation rift valley section within Gorongosa National Park in Sofala Province, Central Mozambique. The single specimen was collected from a dry sand forest with scattered, emergent trees including Baobab trees (Fig. 11). Several bags of sandy soil under a thin layer of leaves on the surface were collected and brought back to the lab to be hand sorted while ants were still alive. A single specimen covered in sand was collected (Fig. 13). Unfortunately, repeated trips to the same locality and microhabitat did not produce any additional specimens. The species is also known from the Tete region in northern Mozambique, where a single worker and a dealate queen were found at different localities (separated by 24 km) north of Moatize. In contrast to the holotype, both of these additional specimens were found under stones in rocky outcrops in open undifferentiated Zambezian woodland.

#### Taxonomic notes.

*Proceratium carri* displays a character combination that renders it easily identifiable within the *Proceratium arnoldi* clade. The most conspicuous difference is the lack of maculae on the vertexal margins of the head, which are present in all examined specimens of the other six species. Nonetheless, this character can be challenging to detect sometimes, especially under low magnifications or with the wrong lighting. An additional character unique to *Proceratium carri* is the relatively long abdominal segment IV, which is around 1.6 times longer than abdominal tergite III (ASI 156–159), whereas it is much shorter in the other four species, around 1.0 to 1.3 times longer (ASI 102–132). Furthermore, *Proceratium carri* has a comparatively thin head in full-face view (CI 85–86), which is shared by *Proceratium arnoldi* (CI 85–87), but strongly contrasts with the head shape of the other five species (CI 91–95).

#### Variation.

We cannot observe any significant intraspecific variation between the two known workers of this species. The only difference is that the specimen from Gorongosa is of darker colour than the one from Moatize.

### Proceratium lunatum

Taxon classificationAnimaliaHymenopteraFormicidae

Terron, 1981

**Figure 14.**
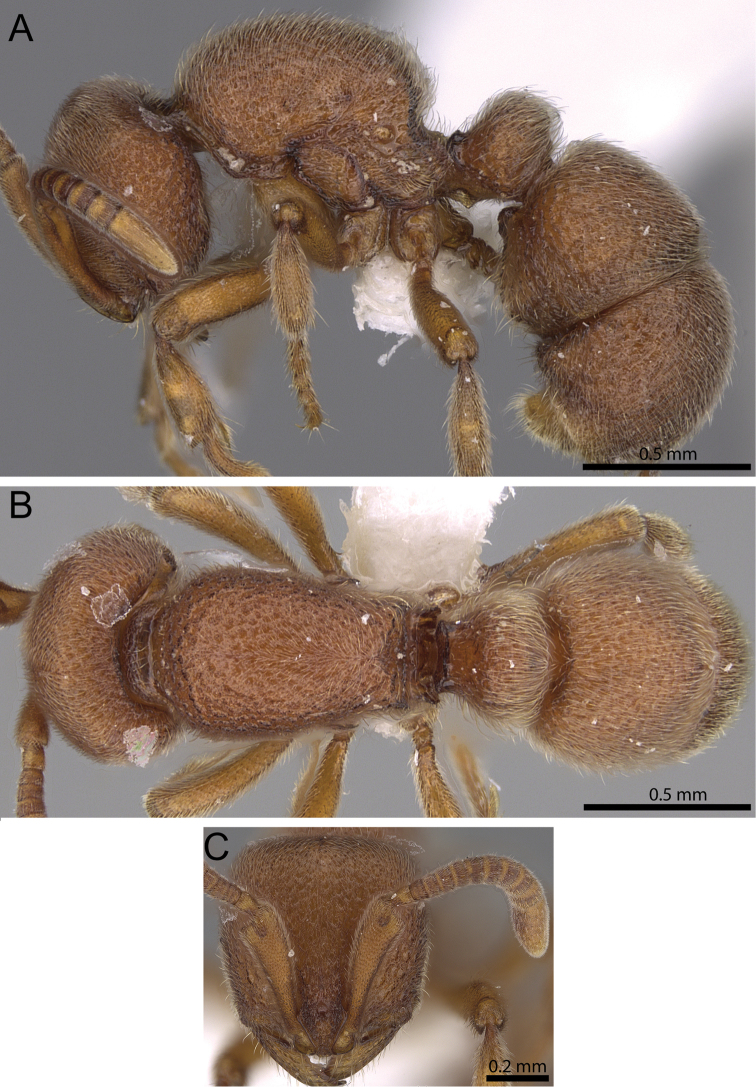
*Proceratium lunatum* worker (CASENT0914280) (Michele Esposito 2014). **A** Body in profile **B** Body in dorsal view **C** head in full-face view.

Figs 3F , 5B , 5E , 6C , 8C , 8D , 14A , 14B , 14C , 18

Proceratium lunatum Terron, 1981: 96. [see also Baroni Urbani and de Andrade 2003: 290]

#### Type material.

**Holotype**, pinned worker, CAMEROON, Arboretum de Mbalmayo (51 km S of Yaounde), no. 1579, 17.III.1968, (*G. Terron*) (CIRAD). **Paratypes**, one pinned paratype worker from CAMEROON, Kala (18 km W Yaounde), Ve Berlese, sp. 1, tamisage terre et terreau, 16.V.1974, (*G. Terron*) (CIRAD); two pinned paratype workers from CAMEROON, Mbalmayo, no. 1759, 17.III.1968 (*G. Terron*) (BMNH: CASENT0902426; MNHN); one pinned paratype worker from CAMEROON, UO Bikok, tamisage terre et terreau, 19.III.1974, (*G. Terron*) (MHNG: CASENT0914221).

#### Non-type material.

CAMEROON: Sud-Ouest, Korup N. P., 6.9 km 317°NW Mundemba, 5.016 N, 8.864 E, 110 m, rainforest, 19.IV.2000 (*B.L. Fisher*) (CASC: CASENT0005926); GABON: Woleu-Ntem, 31.3 km 108°ESE Minvoul, 2.08N, 12.40667E, 600 m, rainforest, 11.II.1998 (*B.L. Fisher*) (CASC: CASENT0914280); UGANDA: Western Uganda, Kabarole, Kibale National Park, Kanyawara Biological Station, 0.56437N, 30.36059E, 1520 m, moist evergreen forest, 8.–11.VIII.2012 (*B.L. Fisher et al.*) (CASC: CASENT0355483).

#### Diagnosis.

The following character combination distinguishes *Proceratium lunatum* from the other Afrotropical members of the *Proceratium arnoldi* clade: eyes strongly reduced (OI 3–5), usually consisting of a single ommatidium, rarely more; head slightly longer than broad (CI 92–95); maculae on vertexal angles of head well developed and conspicuous; mesopleurae weakly to moderately inflated posteriorly; petiolar node high nodiform, anteroposteriorly compressed, with anterior face relatively straight; petiole in dorsal view between 1.2 to 1.3 times wider than long DPeI 122–129; ventral process of petiole lamelliform and approximately rectangular, lamella not significantly pointing anteriorly nor posteriorly; abdominal segment IV between 1.0 to 1.2 times longer than abdominal segment III (ASI 104–118); head, mesosoma and petiole with mat of short decumbent to subdecumbent pubescence only, without any longer, fine suberect to erect hairs.

#### Worker measurements

**(N=8).** TL 2.81–3.43; EL 0.02–0.04; SL 0.46–0.60; HL 0.73–0.84; HLM 0.86–0.96; HW 0.68–077; WL 0.84–1.00; HFeL 0.52–0.63; HTiL 0.41–0.51; HBaL 0.31–0.42; PeL 0.27–0.30; PeW 0.35–0.38; DPeI 122–129; LT3 0.40–0.57; LS4 0.14–0.23; LT4 0.43–0.60; OI 3–5; CI 92–95; SI 63–71; IGR 0.33–0.38; ASI 104–118.

#### Worker description.

In full-face view head slightly longer than broad (CI 92–95), sides weakly convex, vertex flat to weakly convex. Clypeus medially reduced, its anterior margin subconvex to slightly triangular, only slightly protruding anteriorly, not surrounding the antennal sockets and not medially impressed, antennal socket with broad torulus. Frontal carinae relatively short and widely separated, not converging medially and strongly diverging posteriorly, partially covering antennal insertions; frontal carinae conspicuously raised on their anterior half, much less posteriorly. Eyes very small, consisting one to three or four weak ommatidia (OI 3–5) and located on mid line of head. Mandibles elongate-triangular; masticatory margin of mandibles with four relatively small teeth/denticles, decreasing in size from larger apical tooth to basal denticle. Mesosoma in profile convex and approximately as long as the maximum head length including mandibles. Lower mesopleurae with well impressed sutures, no other sutures developed on lateral or dorsal mesosoma; mesopleurae weakly to moderately inflated posteriorly; propodeum in profile armed with small, blunt teeth, propodeal lobes well developed, lamellate, rounded to subtriangular and blunt; declivitous face of propodeum between teeth and lobes noticeably concave; in posterodorsal view sides of propodeum separated from declivitous face by margin connecting propodeal lobes and propodeal teeth. Legs slender and elongate; pro- and mesotibiae with pectinate spurs; calcar of strigil without basal spine. Petiolar node in profile high, blocky nodiform, anterior face of petiole relatively straight, anterior and posterior faces approximately parallel, dorsum of node flat to weakly convex; petiole in dorsal view between 1.2 and 1.3 times wider than long (DPeI 122–129), petiolar node in dorsal view clearly much broader than long; ventral process of petiole lamelliform and approximately rectangular, lamella not significantly pointing anteriorly nor posteriorly. In dorsal view abdominal segment III anteriorly broader than petiole; its sides diverging posteriorly; dorsum of abdominal tergum III with posteromedial, very conspicuous, semitransparent, raised bulla below the integument; abdominal sternite III anteromedially with a marked subtriangular projection. Constriction between abdominal segment III and IV conspicuously impressed. Abdominal segment IV strongly recurved (IGR 0.33–0.38), conspicuously rounded on its curvature, especially posteriorly, abdominal tergum IV between 1.0 and 1.2 times longer than abdominal segment III (ASI 104–118); large, semitransparent and circular bulla situated posteromedially on abdominal tergum IV; remaining abdominal tergites and sternites relatively inconspicuous and curved ventrally. Whole body covered with dense mat of relatively short, decumbent to subdecumbent pubescence without abundant, much longer, suberect to erect, long, fine, standing hairs. Mandibles longitudinally rugose; most of body irregularly foveolate and/or granulate, sometimes more weakly developed on cephalic dorsum and anterior part of abdominal tergum IV, posteroventral part of abdominal tergum IV with conspicuous irregular rugosity; inflated, posterior part of mesopleura and declivitous face of propodeum unsculptured, smooth and shining. Head, mesosoma (excluding posteriorly inflated part of mesopleurae), postpetiole and remaining abdominal segments of light brown to brown colour, mandibles, inflated part of mesopleurae and legs always of lighter brown colour.

#### Distribution and ecology.

*Proceratium lunatum* is known to occur in Cameroon, Gabon and Uganda (Fig. 18) where it was collected in rainforests at elevations ranging from 110 to 1520 m. The known specimens were either collected from within the soil or sifted litter suggesting that *Proceratium lunatum* is a predominantly hypogaeic species.

#### Taxonomic notes.

The recognition of *Proceratium lunatum* within the *Proceratium arnoldi* clade is fairly easy and straightforward. The relatively broad head in full-face view (CI 92–95) groups it together with *Proceratium burundense*, *Proceratium nilo*, *Proceratium sali* and *Proceratium sokoke* (CI 91–95) while it separates it from *Proceratium arnoldi* and *Proceratium carri* that have thinner heads (CI 85–87). The lack of long, standing pilosity on top of a dense mat of much shorter pubescence distinguishes *Proceratium lunatum* from *Proceratium carri*, *Proceratium nilo*, *Proceratium sali* and *Proceratium sokoke*. The species closest to *Proceratium lunatum* seems to be *Proceratium burundense*. However, both differ in eye size, ventral process of the petiole, and propodeal armament. *Proceratium lunatum* has smaller eyes (OI 3–5) and shorter propodeal teeth than *Proceratium burundense* (OI 8). In addition, the ventral process of the petiole has a very distinct posteroventral corner that strongly projects ventrally, whereas the process of *Proceratium lunatum* is more or less rectangular without a projecting posteroventral corner.

#### Variation.

The *Proceratium lunatum* material from Cameroon and Gabon shows no observable intraspecific variation. The specimen from Uganda, however, displays some noticeable differences. It possesses longer antennal scapes (SI 71 vs. SI 63–66) and is generally larger than the western specimens (TL 3.43 vs. TL 2.81–2.94). Nevertheless, we consider these differences as intraspecific variation. The difference in body size is well within the range of other species, thus not significant, and the longer antennal scape alone is not sufficient to warrant species status. This assessment might change with further material from the eastern parts of the equatorial forest belt, such as Uganda, Rwanda and Kenya, but for the moment we keep all the material listed here as *Proceratium lunatum* as one species.

### Proceratium nilo sp. n.

Taxon classificationAnimaliaHymenopteraFormicidae

http://zoobank.org/6D9A7B7F-46EC-40D1-A34E-27362E42D23D

**Figure 15.**
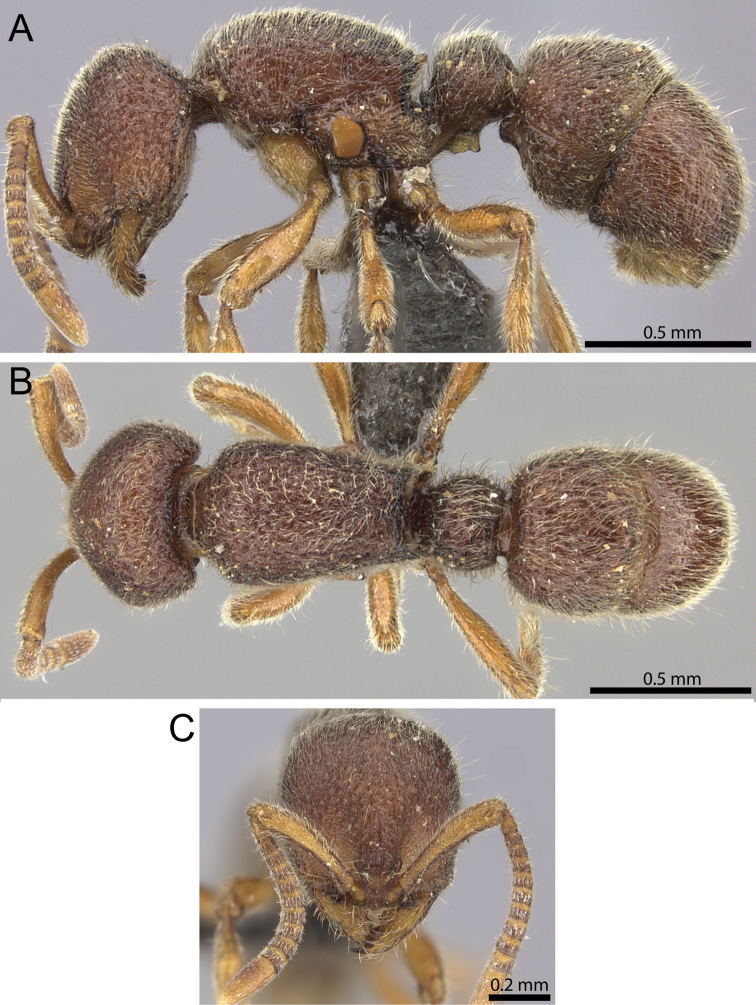
*Proceratium nilo* sp. n. holotype worker (CASENT0235688) (Will Ericson 2011). **A** Body in profile **B** Body in dorsal view **C** head in full-face view.

Figs 4A , 15A , 15B , 15C , 18

#### Type material.

**Holotype**, pinned worker, TANZANIA, Tanga, Korogwe, Nilo Forest Reserve, 4.91456S, 38.67712E, 1006 m, primary forest, collection code CEPF-TZ-4.1, 1.–4.IX.2005 (*P. Hawkes, J. Makwati & R. Mtana*) (SAMC: CASENT0235688).

#### Diagnosis.

*Proceratium nilo* can be distinguished from the other Afrotropical members of the *Proceratium arnoldi* clade by the following combination of characters: eyes absent; head slightly longer than broad (CI 91); maculae on vertexal angles of head well developed and conspicuous; mesopleurae extremely inflated posteriorly; petiolar node in profile relatively low, bluntly rounded nodiform, anterior face of petiole strongly produced anteriorly on lower third and not straight; petiole in dorsal view between 1.1 and 1.2 times wider than long (DPeI 115); ventral process of petiole well developed, lamelliform and rectangular, lamella not pointed anteriorly nor posteriorly; abdominal segment IV around as long as abdominal segment III (ASI 102); head, mesosoma and petiole with numerous long, fine, suberect to erect hairs on top of dense mat of much shorter decumbent to subdecumbent pubescence.

#### Worker measurements

**(N=1).** TL 3.31; EL n.a. (eyes absent); SL 0.56; HL 0.82; HLM 0.99; HW 0.75; WL 0.97; HFeL 0.60; HTiL 0.51; HBaL 0.40; PeL 0.34; PeW 0.39; DPeI 115; LT3 0.50; LS4 0.20; LT4 0.51; OI 0; CI 91; SI 68; IGR 0.39; ASI 102.

#### Worker description.

In full-face view head slightly longer than broad (CI 91), sides weakly convex, head not gently diverging posteriorly, vertex weakly convex. Clypeus medially reduced, its anterior margin convex to slightly triangular, only slightly protruding anteriorly, not surrounding the antennal sockets and not medially impressed, antennal socket with broad torulus. Frontal carinae relatively short and widely separated, not converging medially and strongly diverging posteriorly, partially covering antennal insertions; frontal carinae conspicuously raised on their anterior half, much less posteriorly. Eyes absent (OI 0). Mandibles elongate-triangular; masticatory margin of mandibles with four relatively small teeth/denticles, decreasing in size from larger apical tooth to basal denticle. Mesosoma weakly to moderately convex in profile and approximately as long as the maximum head length including mandibles. Lower mesopleurae with well impressed sutures, no other sutures developed on lateral or dorsal mesosoma; mesopleurae extremely inflated posteriorly; propodeum in profile armed with small, pointed teeth, propodeal lobes well developed, lamellate, rounded and blunt; declivitous face of propodeum between teeth and lobes noticeably concave; in posterodorsal view sides of propodeum separated from declivitous face by margin connecting propodeal lobes and propodeal teeth. Legs slender and elongate; pro- and mesotibiae with pectinate spurs; calcar of strigil without basal spine. Petiolar node in profile relatively low, bluntly rounded nodiform, anterior face of petiole strongly produced anteriorly on lower third and not straight, posterior face approximately straight, anterior and posterior faces not parallel, dorsum of node weakly rounded; petiole in dorsal view between 1.1 and 1.2 times wider than long (DPeI 115), petiolar node in dorsal view clearly much broader than long; ventral process of petiole well developed, lamelliform and rectangular, lamella not pointed anteriorly nor posteriorly. In dorsal view abdominal segment III anteriorly broader than petiole; its sides diverging posteriorly; dorsum of abdominal tergum III with posteromedial, very conspicuous, semitransparent, flat bulla below the integument; abdominal sternite III anteromedially with a marked subtriangular projection. Constriction between abdominal segment III and IV conspicuously impressed. Abdominal segment IV strongly recurved (IGR 0.39), conspicuously rounded on its curvature, especially posteriorly, abdominal tergum IV approximately as long as abdominal segment III (ASI 102); large, semitransparent and semicircular bulla situated posteromedially on abdominal tergum IV; remaining abdominal tergites and sternites relatively inconspicuous and curved ventrally. Whole body covered with dense mat of relatively short, decumbent to subdecumbent pubescence, and most of body with moderately abundant, much longer (several times longer than pubescence), suberect to erect, fine, standing hairs. Mandibles longitudinally rugose; most of body irregularly foveolate and/or granulate, sculpture best developed on cephalic dorsum, moderately so on mesosoma and petiole, especially weak, almost smooth, on most on anterior third of abdominal tergum IV, posterior third of abdominal tergum IV with conspicuous, longitudinal, irregular rugosity; inflated, posterior part of mesopleura and declivitous face of propodeum unsculptured, smooth and shining. Head, mesosoma (excluding posteriorly inflated part of mesopleurae), postpetiole and remaining abdominal segments of brown colour, mandibles, inflated part of mesopleurae and legs yellowish to light brown.

#### Etymology.

The name of the new species is derived from the type locality, the Nilo Forest Reserve in Tanzania. The species epithet is a noun in apposition and thus invariant.

#### Distribution and ecology.

Like several other species of the clade, *Proceratium nilo* is only known from a singleton holotype collected in the Nilo Forest Reserve in the Tanga region of northeast Tanzania (Fig. 18). Nilo covers an area of 5366 ha and, although the 9048 ha Amani Nature Reserve is significantly larger, Nilo is the largest of the 14 forest reserves in the East Usambara mountain range. The forest is largely undisturbed with a dense canopy cover (estimated at 90–95%) and little evidence of logging. Altitude within the reserve ranges from approximately 340 to 1500 m; the area surveyed was near the middle of this range at approximately 1000 m. The soil along the 230 m transect sampled varied from moist loamy sand to sandy clay loam (hand soil texture classification) and roughly 80% covered by an approximately 1 cm thick layer of leaf litter, with deeper accumulations in places. The single *Proceratium nilo* specimen was collected in pitfall trap 18 of 24 placed along the transect, and no further details of its microhabitat preferences can be determined.

#### Taxonomic notes.

*Proceratium nilo* is a fairly conspicuous member of the clade, and possesses a unique character combination allowing an easy identification. The most noticeable difference is the total lack of eyes, which are present in all the other species of the clade. Not considering the eyes, the shape of the petiolar node groups *Proceratium nilo* with *Proceratium sokoke* while it separates it from *Proceratium arnoldi*, *Proceratium burundense*, *Proceratium carri*, *Proceratium lunatum* and *Proceratium sali*. In the latter five the node is high nodiform, anteroposteriorly compressed and with the anterior face relatively straight, whereas the node shape of *Proceratium nilo* and *Proceratium sokoke* is relatively low, bluntly rounded nodiform with the anterior face strongly produced anteriorly on lower third. Despite the clear separation based on the presence/absence of the eyes, *Proceratium nilo* is morphologically very close to *Proceratium sokoke*. Indeed, the only significant difference is eye development, and for a short while we considered to lump them both under the same species name. However, the examination of many more species of *Proceratium* led us to refrain from doing so. As it seems, the presence or absence of eyes, as well as their specific development, is species-specific in the genus, which supports the separation into two species. Also, there are a few more differences. *Proceratium sokoke* has a longer abdominal tergum IV in relation to abdominal segment III (ASI 125) compared to *Proceratium nilo* (ASI 102). In addition, the head of *Proceratium nilo* does not significantly broaden posteriorly while the head of *Proceratium sokoke* does so. However, based on the very limited material this could just be within a normal species-specific range. The two-species hypothesis is also supported by different habitat preferences (littoral, mixed dry forest at a very low elevation vs. submontane, primary rainforest at a medium elevation). Future sampling in East Africa might provide additional evidence for their heterospecificity or not (if eye development turns out to be variable within species), but for the moment we prefer to describe *Proceratium nilo* and *Proceratium sokoke* as easily identifiable species and make them both available to the taxonomic community.

#### Variation.

Since this species is known only from the holotype there is no available information about intraspecific variation.

### Proceratium sali sp. n.

Taxon classificationAnimaliaHymenopteraFormicidae

http://zoobank.org/F9D11EEE-59A5-44E9-9837-0081F4860F68

**Figure 16.**
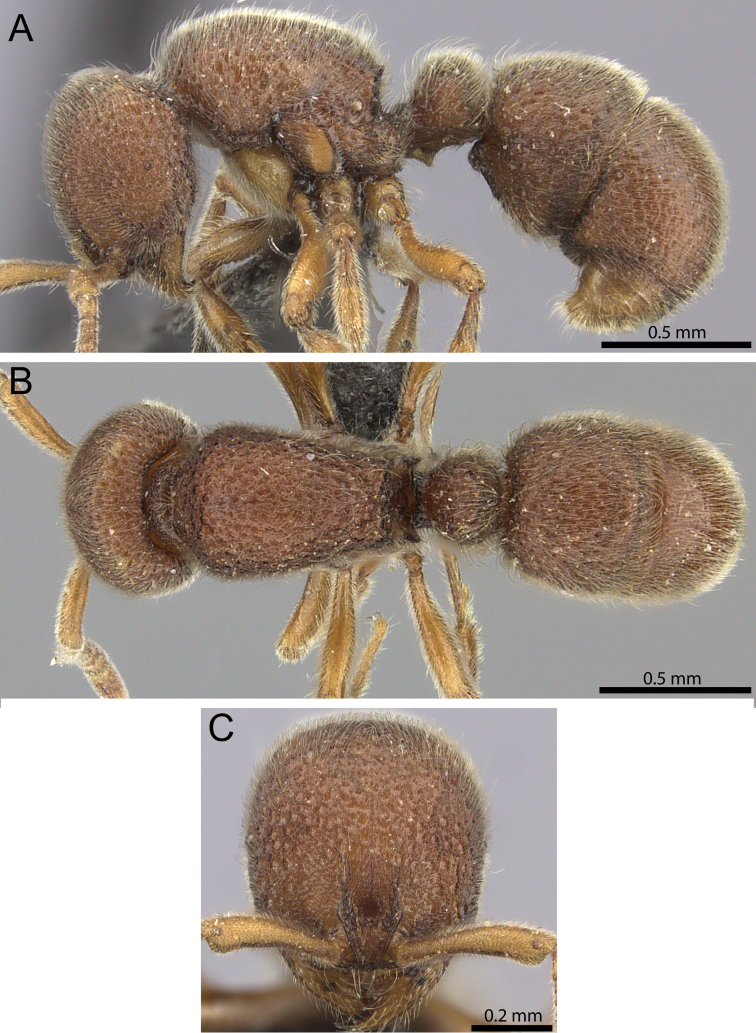
*Proceratium sali* sp. n. holotype worker (CASENT0235689) (Will Ericson 2011). **A** Body in profile **B** Body in dorsal view **C** head in full-face view.

Figs 3C , 6B , 7B , 16A , 16B , 16C , 18

#### Type material.

**Holotype**, pinned worker, TANZANIA, Morogoro, Ulanga, Sali Forest Reserve, 8.94497S, 36.67261E, 1150 m, primary forest, collection code CEPF-TZ-9.1, 17.–20.X.2007 (*P. Hawkes, M. Bhoke & U. Richard*) (SAMC: CASENT0235689).

#### Diagnosis.

The following character combination separates *Proceratium sali* from the other Afrotropical members of the *Proceratium arnoldi* clade by the following combination of characters: eyes very small, consisting of three to four weak ommatidia (OI 5); CI 94; maculae on vertexal angles of head well developed and conspicuous; petiolar node high nodiform, anteroposteriorly compressed, with anterior face relatively straight; petiole in dorsal view between 1.1 and 1.2 times wider than long (DPeI 116); ventral process of petiole well developed, lamelliform and rectangular, lamella not pointed anteriorly nor posteriorly; abdominal segment IV around 1.1 times longer than abdominal segment III (ASI 108); head, mesosoma and petiole with numerous long, fine, suberect to erect hairs on top of dense mat of much shorter decumbent to subdecumbent pubescence.

#### Worker measurements

**(N=1).** TL 3.35; EL 0.04; SL 0.55; HL 0.81; HLM 0.94; HW 0.76; WL 1.00; HFeL 0.62; HTiL 0.53; HBaL 0.41; PeL 0.31; PeW 0.36; DPeI 116; LT3 0.53; LS4 0.19; LT4 0.57; OI 5; CI 94; SI 68; IGR 0.33; ASI 108.

#### Worker description.

In full-face view head slightly longer than broad (CI 94), sides and vertex moderately convex. Clypeus medially reduced, its anterior margin convex to slightly triangular, only slightly protruding anteriorly, not surrounding the antennal sockets and not medially impressed, antennal socket with broad torulus. Frontal carinae relatively short and widely separated, not converging medially and strongly diverging posteriorly, partially covering antennal insertions; frontal carinae conspicuously raised on their anterior half, much less posteriorly. Eyes very small, consisting of three to four faint ommatidia (OI 5) and located on mid line of head. Mandibles elongate-triangular; masticatory margin of mandibles with four relatively small teeth/denticles, decreasing in size from larger apical tooth to basal denticle. Mesosoma weakly to moderately convex in profile and weakly longer than maximum head length including mandibles. Lower mesopleurae with well impressed sutures, no other sutures developed on lateral or dorsal mesosoma; mesopleurae extremely inflated posteriorly; propodeum in profile armed with very small, pointed teeth, propodeal lobes well developed, triangular and blunt; declivitous face of propodeum between teeth and lobes noticeably concave; in posterodorsal view sides of propodeum separated from declivitous face by margin connecting propodeal lobes and propodeal teeth. Legs slender and elongate; pro- and mesotibiae with pectinate spurs; calcar of strigil without basal spine. Petiolar node in profile high, blocky nodiform, anterior face of petiole relatively straight, anterior and posterior faces approximately parallel, dorsum of node flat to weakly convex; petiole in dorsal view between 1.1 and 1.2 times wider than long (DPeI 116), petiolar node in dorsal view clearly much broader than long; ventral process of petiole well developed, lamelliform and rectangular, lamella not pointed anteriorly nor posteriorly. In dorsal view abdominal segment III anteriorly broader than petiole; its sides diverging posteriorly; dorsum of abdominal tergum III with posteromedial, very conspicuous, semitransparent, flat bulla below the integument; abdominal sternite III anteromedially with a marked subtriangular projection. Constriction between abdominal segment III and IV conspicuously impressed. Abdominal segment IV strongly recurved (IGR 0.33), conspicuously rounded on its curvature, especially posteriorly, abdominal tergum IV only less than 1.2 times longer than abdominal segment III (ASI 116); large, semitransparent and semicircular bulla situated posteromedially on abdominal tergum IV; remaining abdominal tergites and sternites relatively inconspicuous and curved ventrally. Whole body covered with dense mat of relatively short, decumbent to subdecumbent pubescence; most of body with moderately abundant, much longer (several times longer than pubescence), suberect to erect, fine, standing hairs. Mandibles longitudinally rugose; most of body irregularly foveolate and/or granulate, sculpture best developed on cephalic and mesosomal dorsum, less so on mesosoma and especially weak on most of relatively smooth and shining abdominal tergum IV; inflated, posterior part of mesopleura and declivitous face of propodeum unsculptured, relatively smooth and shining. Head, mesosoma (excluding posteriorly inflated part of mesopleurae), postpetiole and remaining abdominal segments of brown colour, mandibles, inflated part of mesopleurae and legs yellowish to light brown.

#### Etymology.

The name of the new species is derived from the type locality, the Sali Forest Reserve in Tanzania. The species epithet is a noun in apposition and thus invariant.

#### Distribution and ecology.

*Proceratium sali* is only known from the Sali Forest Reserve in the Morogoro region of south-central Tanzania (Fig. 18). Sali covers an area of 1072 ha and is the largest of the seven reserves in the Mahenge mountain range. The forest is largely undisturbed with little evidence of logging and a fairly dense canopy cover (estimated at 80–90%). Altitude within the reserve ranges from approximately 1150 to 1480 m and the area surveyed was at the lower end of this range. The soil along the 230 m transect surveyed was moist sandy clay loam (hand soil texture classification) and approximately 90% covered by a 2–3 cm thick layer of leaf litter. The single *Proceratium sali* specimen was collected in pitfall trap 16 of 24 placed along the transect, and no further details of its microhabitat preferences can be determined.

#### Taxonomic notes.

*Proceratium sali* shares a thicker head in full-face view (CI 94) with *Proceratium burundense*, *Proceratium lunatum*, *Proceratium nilo* and *Proceratium sokoke* (CI 91–95), which contrasts with the thinner head of *Proceratium arnoldi* and *Proceratium carri* (85–87). In addition, *Proceratium sali* (as well as *Proceratium carri*, *Proceratium nilo*, *Proceratium sali* and *Proceratium sokoke*) possesses numerous long, fine, standing hairs on top of a mat of much shorter pubescence distinguishing it from *Proceratium arnoldi*, *Proceratium lunatum* and *Proceratium burundense* that lack this type of pilosity. The two species most similar to *Proceratium sali* are *Proceratium nilo* and *Proceratium sokoke*, but the latter two have a lower, less compressed petiolar node with the anterior face strongly produced anteriorly on lower third. This contrasts strongly with the node of *Proceratium sali* that is high nodiform and more compressed with a straight anterior face.

#### Variation.

As for *Proceratium burundense*, *Proceratium nilo* and *Proceratium sokoke*, *Proceratium sali* is also only known from a singleton holotype, which does not allow any conclusions on intraspecific variation.

### Proceratium sokoke sp. n.

Taxon classificationAnimaliaHymenopteraFormicidae

http://zoobank.org/23A889EA-F16E-4142-ACC6-6AA0F6D5B382

**Figure 17.**
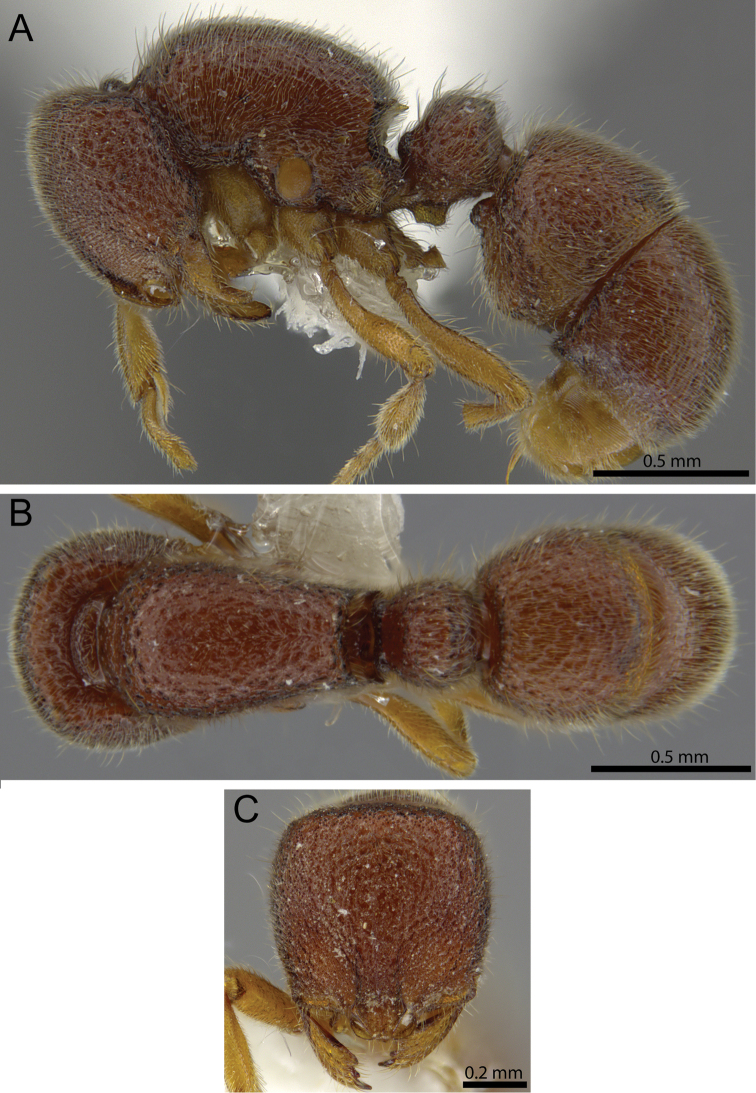
*Proceratium sokoke* sp. n. holotype worker (MCZ-ENT00520482). **A** Body in profile **B** Body in dorsal view **C** head in full-face view.

**Figure 18.**
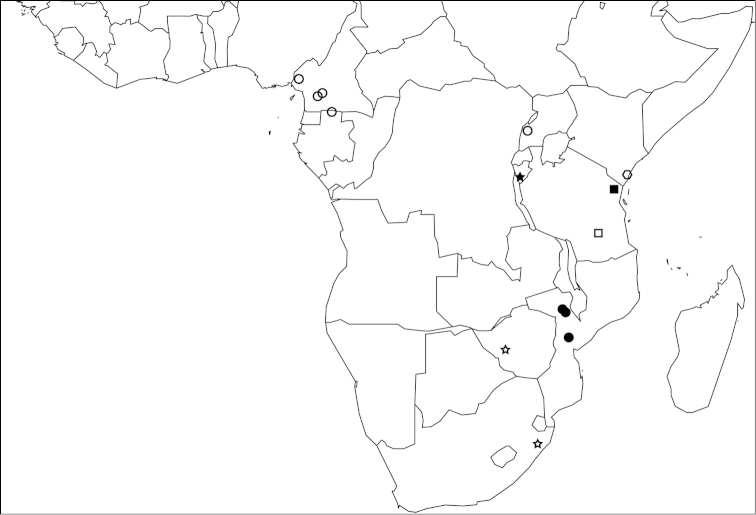
Map of sub-Saharan Africa showing the known distribution ranges of the seven Afrotropical members of the *Proceratium arnoldi* clade: *Proceratium arnoldi* (empty star), *Proceratium burundense* (filled star), *Proceratium carri* (filled circle), *Proceratium lunatum* (empty circle), *Proceratium nilo* (filled square), *Proceratium sali* (empty square), *Proceratium sokoke* (empty hexagon).

Figs 1D , 3B , 6A , 7A , 17A , 17B , 17C , 18

#### Type material.

**Holotype**, pinned worker, KENYA, Coastal Province, Arabuko Sokoke Forest, 18°51'72"S, 39°56'26.6"E, 136 m, undisturbed and protected mixed forest, Winkler leaf litter extraction, collection code FHG00206, VI.2009 (*F. Hita Garcia & G. Fischer*) (MCZ: MCZ-ENT00520482).

#### Diagnosis.

*Proceratium sokoke* differs from the other Afrotropical members of the *Proceratium arnoldi* clade by the following character combination: eyes strongly reduced, consisting of a single ommatidium (OI 4); CI 92; maculae on vertexal angles of head well developed and conspicuous; petiolar node in profile relatively low, bluntly rounded nodiform, anterior face of petiole strongly produced anteriorly on lower third and not straight; petiole in dorsal view between 1.1 to 1.2 times wider than long (DPeI 115); ventral process of petiole well developed, lamelliform and rectangular, lamella not pointed anteriorly nor posteriorly; abdominal segment IV around 1.25 times longer than abdominal segment III (ASI 125); head, mesosoma and petiole with numerous long, fine, suberect to erect hairs on top of dense matt of much shorter decumbent to subdecumbent pubescence.

#### Worker measurements

**(N=1).** TL 2.47; EL 0.03; SL n.a.; HL 0.72; HLM 0.87; HW 0.66; WL 0.86; HFeL n.a.; HTiL n.a.; HBaL n.a.; PeL 0.30; PeW 0.35; DPeI 115; LT3 0.44; LS4 0.14; LT4 0.55; OI 4; CI 92; SI n.a.; IGR 0.25; ASI 125.

#### Worker description.

[Note: the holotype is partly damaged: antennae, one foreleg, one midleg and one hindleg missing, remaining hindleg broken at level of tibia].

Head longer than broad (CI 92), sides weakly convex, cephalic dorsum broader posteriorly than anteriorly; vertex in full-face view flat to weakly convex. Clypeus medially reduced, its anterior margin convex to slightly triangular, only slightly protruding anteriorly, not surrounding the antennal sockets and not medially impressed, antennal socket with broad torulus. Frontal carinae relatively short and widely separated, not converging medially and strongly diverging posteriorly, partially covering antennal insertions; frontal carinae conspicuously raised on their anterior two thirds, much less posteriorly. Eyes very small (OI 4), consisting only of one ommatidium and located on mid line of head. Mandibles elongate-triangular; masticatory margin of mandibles with four relatively small teeth/denticles, decreasing in size from larger apical tooth to basal denticle. Mesosoma weakly to moderately convex in profile and approximately as long as the maximum head length including mandibles. Lower mesopleurae with well impressed sutures, no other sutures developed on lateral or dorsal mesosoma; mesopleurae extremely inflated posteriorly; propodeum in profile armed with small, pointed teeth, propodeal lobes well developed, lamellate, rounded and blunt; declivitous face of propodeum between teeth and lobes noticeably concave; in posterodorsal view sides of propodeum separated from declivitous face by margin connecting propodeal lobes and propodeal teeth. Legs slender and elongate; pro- and mesotibiae with pectinate spurs; calcar of strigil without basal spine. Petiolar node in profile relatively low, bluntly rounded nodiform, anterior face of petiole strongly produced anteriorly on lower third and not straight, posterior face approximately straight, anterior and posterior faces not parallel, dorsum of node weakly rounded; petiole in dorsal view between 1.1 to 1.2 times wider than long (DPeI 115), petiolar node in dorsal view clearly much broader than long; ventral process of petiole well developed, lamelliform and rectangular, lamella not pointed anteriorly nor posteriorly. In dorsal view abdominal segment III anteriorly broader than petiole; its sides diverging posteriorly; dorsum of abdominal tergum III with posteromedial, very conspicuous, semitransparent, flat bulla below the integument; abdominal sternite III anteromedially with a marked subtriangular projection. Constriction between abdominal segment III and IV conspicuously impressed. Abdominal segment IV strongly recurved (IGR 0.25), conspicuously rounded on its curvature, especially posteriorly, abdominal tergum IV around 1.25 times longer than abdominal segment III (ASI 125); large, semitransparent and semicircular bulla situated posteromedially on abdominal tergum IV; remaining abdominal tergites and sternites relatively inconspicuous and curved ventrally. Whole body covered with dense matt of relatively short, decumbent to subdecumbent pubescence; most of body with moderately abundant, much longer (several times longer than pubescence), suberect to erect, long, fine, standing hairs. Mandibles longitudinally rugose; most of body irregularly foveolate and/or punctate, sculpture best developed on cephalic dorsum, less so on mesosoma and especially weak on most of relatively smooth and shining abdominal tergum IV; inflated, posterior part of mesopleura and declivitous face of propodeum also only very weakly sculptured and relatively smooth and shining. Head, mesosoma (excluding posteriorly inflated part of mesopleurae), postpetiole and remaining abdominal segments of brown colour, mandibles, inflated part of mesopleurae and legs yellowish to light brown.

#### Etymology.

The name of the new species is inspired by the type locality, the Arabuko Sokoke Forest in Coastal Kenya. The forest is the last larger remnant of the Coastal Forests of Eastern Africa in Kenya and hosts a unique fauna and flora. The species epithet is a noun in apposition and thus invariant.

#### Distribution and ecology.

*Proceratium sokoke* is only known from the type locality, the Arabuko Sokoke Forest in Kenya, which is a tropical dry forest adjacent to the Indian Ocean coast (Fig. 18). As for most of its congeners, the natural history of this species is completely unknown. The holotype was collected from a leaf litter sample in a mixed forest habitat. Unfortunately, *Proceratium sokoke* was only found in that one leaf litter sample and could not be recollected in the remaining 180 litter samples from Arabuko Sokoke, which means that it is either very rare or lives deep in the soil. With the background of the biology of the genus in general, we consider the latter most likely.

#### Taxonomic notes.

The identification of *Proceratium sokoke* is straightforward within the *Proceratium arnoldi* clade. *Proceratium nilo* and *Proceratium sokoke* are the only species of the *Proceratium arnoldi* clade in which the petiolar node in profile does not have a straight anterior face; instead the lower third is produced anteriorly. In the other five species the anterior face is conspicuously straight. *Proceratium nilo* is relatively similar to *Proceratium sokoke*, but the latter has eyes that are absent in the first. Not considering presence/absence of eyes, both species could be seen as conspecific. As discussed in detail in the taxonomic notes section for *Proceratium nilo*, we prefer to treat them as heterospecific in this study since eye development appears to be relatively stable within species of *Proceratium*. In addition to petiolar node shape, *Proceratium sokoke* (CI 92) has a thicker head than *Proceratium arnoldi* and *Proceratium carri* (CI 85–87), and its smaller eyes (O 4) and the rectangular ventral process of the petiole distinguish it from *Proceratium burundense* with its larger eyes (OI 8) and ventral process with an almost spiniform posteroventral corner. Furthermore, the presence of numerous long, fine, suberect to erect hairs on top of a dense mat of much shorter decumbent to subdecumbent pubescence is an additional character that separates *Proceratium sokoke* from *Proceratium arnoldi*, *Proceratium burundense* and *Proceratium lunatum*.

In general it is not recommendable to describe a new species based on a damaged singleton holotype. However, after detailed examination of all the material collected from Arabuko Sokoke, there was no other specimen to be found. In addition, we think that *Proceratium sokoke* is a fairly distinct member of the *Proceratium arnoldi* clade and even without antennae and the missing three and half legs, it can be easily separated from the remainder of the group. Consequently, we prefer to make the species available to science now than to await the discovery of additional material.

#### Variation.

Since the species is only known from the holotype, no information about intraspecific variation exists.

## Supplementary Material

XML Treatment for Proceratium arnoldi

XML Treatment for Proceratium burundense

XML Treatment for Proceratium carri

XML Treatment for Proceratium lunatum

XML Treatment for Proceratium nilo

XML Treatment for Proceratium sali

XML Treatment for Proceratium sokoke

## References

[B1] Arnold G (1915) A monograph of the Formicidae of South Africa. Part I. Ponerinae, Dorylinae. Annals of the South African Museum 14: 1–159

[B2] Baroni Urbani C de Andrade ML (2003) The ant genus *Proceratium* in the extant and fossil record (Hymenoptera: Formicidae). Museo Regionale di Scienze Naturali, Monografie 36: 1–480

[B3] Bolton B (1994) Identification guide to the ant genera of the world. Harvard University Press, Cambridge, 222 pp

[B4] Bolton B (1995) A new general catalogue of the ants of the world. Harvard University Press, Cambridge, 504 pp

[B5] Bolton B (2014) An online catalog of the ants of the world. http://antcat.org [accessed 21 January 2014]

[B6] Brown WL (1958a) Predation of arthropod eggs by the ant genera *Proceratium* and *Discothyrea*. Psyche 64: 115. doi: 10.1155/1957/45849

[B7] Brown WL (1958b) Contributions toward a reclassification of the Formicidae. II. Tribe Ectatommini (Hymenoptera). Bulletin of the Museum of Comparative Zoology 118: 173–362

[B8] Brown WL (1974) A remarkable new island isolate in the genus *Proceratium* (Hymenoptera: Formicidae). Psyche 81: 70–83. doi: 10.1155/1974/90949

[B9] Brown WL (1980) A remarkable new species of *Proceratium*, with dietary and other notes on the genus (Hymenoptera: Formicidae). Psyche 86: 337–346. doi: 10.1155/1979/78461

[B10] Consani M (1851) Formiche dell’Africa Orientale I. Bollettino dell’Istituto di Entomologia della Università degli Studi di Bologna 18: 167–172

[B11] Emery C (1895) Beiträge zur Kenntniss der nordamerikanischen Ameisenfauna. (Schluss). Zoologische Jahrbücher. Abteilung für Systematik, Geographie und Biologie der Tiere 8: 257–360

[B12] Emery C (1897) Formicidarum species novae vel minus cognitae in collectione Musaei Nationalis Hungarici quas in Nova-Guinea, colonia germanica, collegit L. Biró. Természetrajzi Füzetek 20: 571–599

[B13] Evenhuis NL (2014) The insect and spider collections of the world website. http://hbs.bishopmuseum.org/codens [accessed 21 January 2014]

[B14] Fisher BL (2005a) A model for a global inventory of ants: A case study in Madagascar. Proceedings of the California Academy of Sciences 56: 86–97

[B15] Fisher BL (2005b) A new species of *Discothyrea* Roger from Mauritius and a new species of *Proceratium* Roger from Madagascar (Hymenoptera: Formicidae). Proceedings of the California Academy of Sciences 56: 657–667

[B16] Forel A (1913) Ameisen aus Rhodesia, Kapland usw. (Hym.) gesammelt von Herrn G. Arnold, Dr. H. Brauns und Anderen. Deutsche Entomologische Zeitschrift 1913 (Suppl.): 203–225

[B17] Harris RA (1979) A glossary of surface sculpturing. California Department of Food and Agriculture, Bureau of Entomology 28: 1–31

[B18] Hita Garcia F Fisher BL (2011) The ant genus *Tetramorium* Mayr (Hymenoptera: Formicidae) in the Malagasy region – introduction, definition of species groups, and revision of the *T. bicarinatum*, *T. obesum*, *T. sericeiventre* and *T. tosii* species groups. Zootaxa 3039: 1–72

[B19] Hita Garcia F Wiesel E Fischer G (2013) The Ants of Kenya (Hymenoptera: Formicidae)—Faunal overview, first species checklist, bibliography, accounts for all genera, and Discussion on taxonomy and zoogeography. Journal of East African Natural History 101: 127–222. doi: 10.2982/028.101.0201

[B20] Keller RA (2011) A phylogenetic analysis of ant morphology (Hymenoptera, Formicidae) with special reference to the poneromorph subfamilies. Bulletin of the American Museum of Natural History 355: 1–90. doi: 10.1206/355.1

[B21] Leston D (1971) The Ectatommini (Hymenoptera: Formicidae) of Ghana. Journal of Entomology 40: 117–120

[B22] Onoyama K Yoshimura M (2002) The ant genus *Proceratium* (Hymenoptera: Formicidae) in Japan. Entomological Science 5: 29–49

[B23] Perrault GH (1981) *Proceratium deelemani*, nouvelle espèce de Kalimantan. Nouvelle Revue d’Entomologie 11: 189–193

[B24] Roger J (1860) Die *Ponera*-artigen Ameisen. Berliner Entomologische Zeitschrift 4: 278–312

[B25] Roger J (1863) Die neu aufgeführten Gattungen und Arten meines Formiciden-Verzeichnisses nebst Ergänzung einiger früher gegebenen Beschreibungen. Berliner Entomologische Zeitschrift 7: 131–214. doi: 10.1002/mmnd.18630070116

[B26] Snelling RR Cover S (1992) Description of a new *Proceratium* from Mexico (Hymenoptera: Formicidae). Psyche 99: 49–53. doi: 10.1155/1992/61849

[B27] Terron G (1981) Deux nouvelles espèces éthiopiennes pour le genre *Proceratium* (Hym.: Formicidae). Annales de la Faculté des Sciences de Yaoundé 28: 95–103

[B28] Ward PS (1988) Mesic elements in the Western Nearctic ant fauna: taxonomic and biological notes on *Amblyopone*, *Proceratium*, and *Smithistruma* (Hymenoptera: Formicidae). Journal of the Kansas Entomological Society 61: 102–124

[B29] Wilson EO (1955) A monographic revision of the ant genus *Lasius*. Bulletin of the Museum of Comparative Zoology 113: 1–201

[B30] Yoshimura M Fisher BL (2009) A revision of male ants of the Malagasy region (Hymenoptera: Formicidae): Key to genera of the subfamily Proceratiinae. Zootaxa 2216: 1–21

[B31] Yoshimura M Fisher BL (2014) A revision of the ant genus *Mystrium* in the Malagasy region with description of six new species and remarks on *Amblyopone* and *Stigmatomma* (Hymenoptera, Formicidae, Amblyoponinae). ZooKeys 394: 1–99. doi: 10.3897/zookeys.394.6446 24715784 10.3897/zookeys.394.6446 PMC3978267

[B32] Xu Z (2006) Three new species of the ant genera *Amblyopone* Erichson, 1842 and *Proceratium* Roger, 1863 (Hymenoptera: Formicidae) from Yunnan, China. Myrmecologische Nachrichten 8: 151–155

